# USP10 stabilizes BAZ1A to drive tumor stemness via an epigenetic mechanism in head and neck squamous cell carcinoma

**DOI:** 10.1038/s41419-025-07462-x

**Published:** 2025-04-10

**Authors:** Yanni Shi, Jiawei Ding, Xiao Ling, Danfeng Xu, Yan Shen, Xingjun Qin

**Affiliations:** 1 Department of Oral and Maxillofacial Head and Neck Oncology, Shanghai Ninth People’s Hospital, Shanghai Jiao Tong University School of Medicine, National Center for Stomatology, National Clinical Research Center for Oral Diseases, Shanghai Key Laboratory of Stomatology and Shanghai Research Institute of Stomatology, 200011 Shanghai, China; 2 Department of Urology, Ruijin Hospital Affiliated to Shanghai Jiaotong University School of Medicine, 197 Ruijin Road II, Shanghai, 200025 China; 3 Research Center for Experimental Medicine, Ruijin Hospital Affiliated to Shanghai Jiao Tong University School of Medicine, 197 Ruijin Road II, Shanghai, 200025 China

**Keywords:** Cancer, Cell biology

## Abstract

Aberrant epigenetic remodeling events occurred in head and neck squamous cell carcinoma (HNSCC) contribute to tumor stemness and chemotherapy resistance, yet little is known. In this study, we identified that ubiquitin-specific peptidase 10 (USP10) is up-regulated in HNSCC tissues, and high USP10 is associated with poor prognosis of patients. Functionally, USP10 serving as an oncogene potentiates the proliferation and metastasis of HNSCC cells in vitro and in vivo. Mechanistically, USP10 physically interacts with, deubiquitinate, and stabilizes BAZ1A proteins. In addition, BAZ1A complexes with SOX2 to drive the enhancer-promoter interaction and facilitate the recruitment of BRD4, thereby activating the expressions of cancer stem cells (CSCs)-related signature. Therefore, we found that USP10 relied on BAZ1A to enhance HNSCC stemness, progression, and chemotherapy resistance. The pharmacology research implicated that BAZ1A-IN-1, one specific BAZ1A inhibitor, could effectively inhibit HNSCC stemness, distal metastasis, and cisplatin resistance. Together, our study revealed a novel USP10/BAZ1A/stemness axis and one significant therapeutic target for USP10-driven HNSCC.

## Introduction

As reported, head and neck squamous cell carcinoma (HNSCC) is the predominant malignant tumor in the head and neck region [1]. It is composed of various upper respiratory tract tumors and is the seventh most common cancer in the world [2, 3]. In recent decades, multimodal strategies incorporating surgery, radiation therapy, and molecular targeted therapy have yielded substantial clinical advantages for HNSCC patients [4]. Regrettably, a significant percentage of patients will eventually experience recurrence or distant metastases, resulting in a bleak prognosis [5, 6]. No screening strategy has demonstrated efficacy, and meticulous physical examination continues to be the principal method for early detection [7]. Although some oral precancerous lesions (manifested as white or red plaques) progress to invasive cancer, most patients exhibit advanced HNSCC without a prior history of precancerous conditions [8]. Oral HNSCC is usually treated with surgical excision, succeeded by adjuvant radiotherapy or a combination of chemotherapy and radiotherapy (known as radiochemotherapy or chemoradiotherapy) based on disease staging [1]. Chemoradiotherapy has always been the main method for treating throat or laryngeal cancer [9].

Prior research has demonstrated that abnormalities in the ubiquitination modification pathway may influence the effectiveness of chemoradiotherapy in HNSCC [10, 11]. Deubiquitinating enzymes (DUBs) are a category of cysteine proteases or metalloproteinases that can antagonise the function of E3 ligases and are crucial in cell cycle regulation and cancer advancement [12]. The human genome encodes over 100 DUBs, which play significant roles in essential regulatory processes and may thus serve as novel therapeutic targets [13, 14]. Ubiquitin-specific peptidase 10 (USP10) is a highly conserved deubiquitinase widely involved in the initiation and progression of a wide range of cancer types [15–17]. Nonetheless, the role of USP10 in carcinogenesis varies depending on the substrate it interacts with. For example, according to Hong’s research, USP10 promotes the growth of hepatocellular carcinoma via deubiquitinating and stabilizing YAP/TAZ [18].

Another study shows that deubiquitinase USP10 modulates KLF4 stability and inhibits lung carcinogenesis [19]. Consequently, elucidating the pathogenic function of USP10 in HNSCC necessitates mechanistic investigations of its substrates.

Chemotherapy is the initial treatment option for cancer patients [20]. Most patients face the problem of decreased drug efficacy after cancer treatment due to the development of treatment resistance [21]. The resistance to treatment comes from multiple biological mechanisms, including DNA repair, genetic and epigenetic modifications, metabolic reprogramming, enhanced angiogenesis, and tumor microenvironment alterations [22–25]. Therefore, for clinical doctors and researchers who mainly treat metastatic and malignant cases, treatment is a huge challenge, and it is necessary to identify and implement specific target therapies to develop precision medicine [26]. Immune checkpoint inhibitors (ICIs) have initiated a novel phase in cancer therapy, delivering unparalleled therapeutic advantages to patients [27]. Nevertheless, a limited percentage of patients have seen a response, underscoring the imperative for biomarker research to enhance patient selection and combination methods to combat immunological resistance [28]. Cancer stem cells (CSCs) are self-renewing cells that facilitate tumor initiation, progression, and metastasis [29]. More and more evidence shows a significant correlation between stemness and cancer immune evasion and resistance [30].

Herein, we observed that the deubiquitinase USP10 was upregulated in HNSCC tissues and high USP10 was an independent risk factor in HNSCC patients. Whether USP10 is closely related to tumor progression and chemotherapy resistance deserves further investigation in this study.

## Results

### Elevated USP10 expression correlates with poor prognosis of patients with HNSCC

**Fig. 1:**
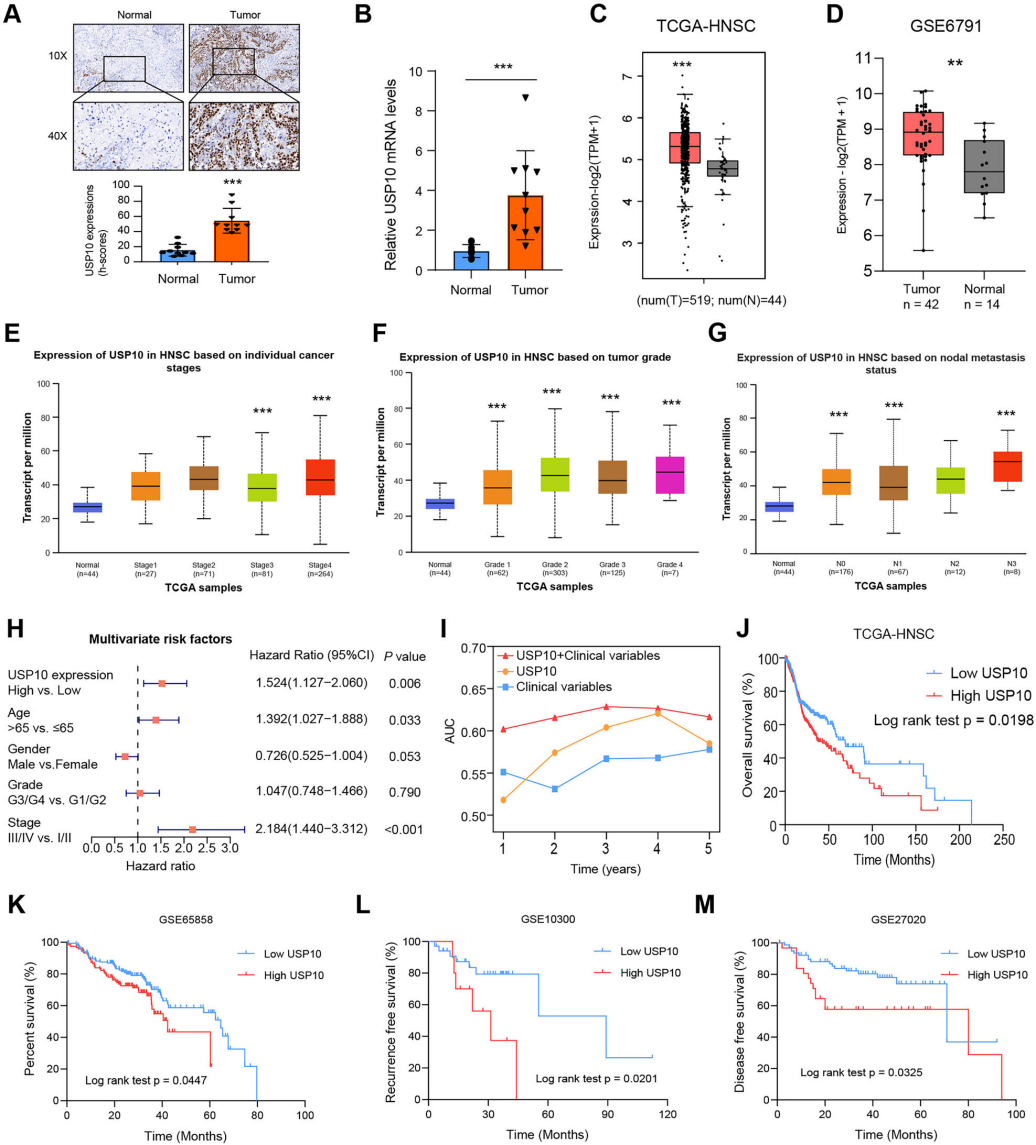
Identification of USP10 as a key oncogene in HNSCC. **A** Immunohistochemistry (IHC) detecting USP10 expressions in HNSCC specimens as well as matched normal tissues. Quantification data was shown below. **B** Quantification of USP10 mRNA levels via RT-qPCR in tumor and normal samples. **C, D** Differential analysis of USP10 mRNA levels in samples derived from TCGA-HNSCC (**C**), and GSE6791 (**D**). **E–G** Kruskal–Wallias test shows the correlations between USP10 levels and clinical characteristics, including tumor stages (**E**), tumor grades (**F**), and nodal metastasis status (**G**). **H** Multivariable analyses were performed in the TCGA-HNSCC cohort. All bars correspond to 95% CIs. **I** The time-dependent receiver operating characteristic (ROC) analysis for the clinical risk score (TNM stage), the USP10 risk score, and the combined USP10 and clinical risk scores in the HNSCC cohort. AUC, the area under the curve. **J** Kaplan–Meier analysis showing the differential overall survival (OS) outcomes in patients with high-, and -low USP10 levels. **K–M** Kaplan–Meier survival curves were generated in other public datasets, including GSE65858 (**K**), GSE10300 (**L**), and GSE27020 (**M**). Data represent the Mean ± SD of at least three independent experiments. *P < 0.05, **P < 0.01, and ***P < 0.001. Differences were tested using an un-paired Student’s *t* test (**B**–**D**), and the log-rank test (**J**–**M**).

To elucidate the role of USP10 in HNSCC, we first detect the USP10 expression levels in 10 HNSCC and paired adjacent normal tissues. Immunohistochemical (IHC) analysis revealed that USP10 protein levels were notably higher in HNSCC tissues (Fig. 1A). RT-qPCR analysis also revealed that USP10 mRNA levels were significantly higher in tumor samples (Fig. 1B). We then queried the public TCGA-HNSCC dataset and found that USP10 is highly expressed in tumor samples than in normal tissues (Fig. 1C). In line with the TCGA results, differential analysis from the samples in GSE6791 (N = 56) also confirmed the higher levels of USP10 in tumors than those in paired normal mucosa tissues (Fig. 1D). Further analysis suggested that high USP10 levels were positively associated with clinical stages, tumor grades, and nodal metastasis status of HNSCC patients (Fig. 1E–G). In addition, univariate Cox regression analysis revealed that clinical stages, tumor grades, and USP10 levels were significantly correlated with 5-year survival in HNSCC patients, and multivariate Cox regression analysis further confirmed that USP10 was a robust independent predictive marker for the prognosis of patients with HNSCC (Fig. 1H). To thoroughly validate the predictive capacity of the USP10 level, we performed a time-dependent receiver operating characteristics curve (ROC) analysis, combining the significant variables of TNM stages and USP10 expressions. We observed that the combination of clinical factors (TNM stage) and USP10 levels contributed much more than either one alone in the HNSCC cohort (Fig. 1I). The Kaplan-Meier analysis of the TCGA-HNSCC cohort suggested that patients with high USP10 expression showed unfavorable overall survival (Fig. 1J). Consistent with this finding, independent GEO datasets (GSE65858, GS10300, GSE27020) further demonstrated that high USP10 is correlated with poorer OS (Fig. 1K–M), showing that USP10 may serve as a hazard biomarker for HNSCC.

### USP10 is required for oncogenic phenotypes of HNSCC

**Fig. 2:**
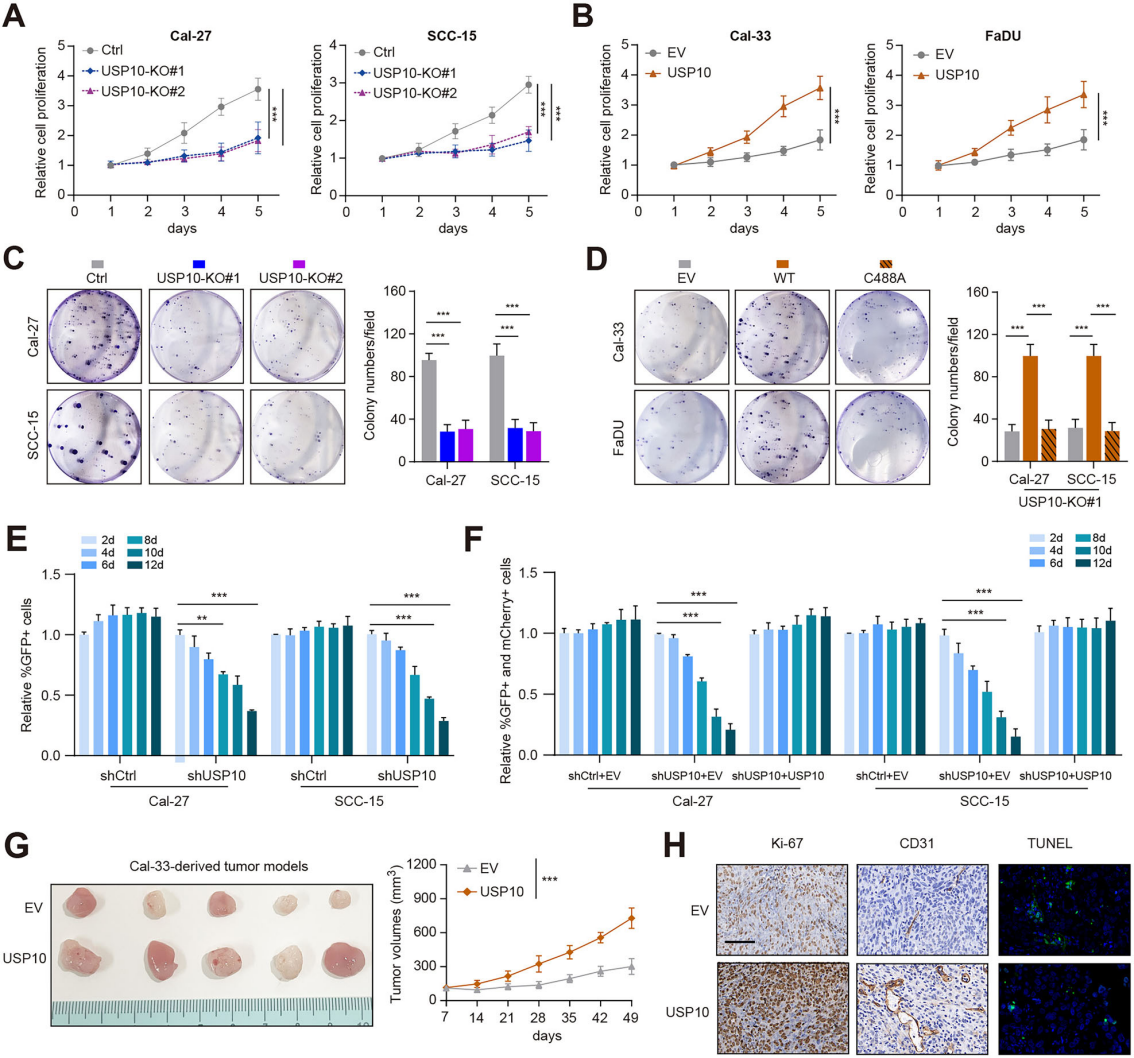
USP10 enhances HNSCC proliferation in vitro and in vivo. **A** CCK-8 assays were used to detect the viability of USP10 knockdown Cal-27 or SCC-15 cells. **B** CCK-8 assays were used to detect the viability of USP10-overexpressing Cal-33 or FaDU cells. **C** Colony formation assays (left) and quantification (right) show the growth capacity of control and USP10-deficient Cal-27 or SCC-15 cells. **D** Colony formation assays (left) and quantification (right) showing the growth capacity of Cal-33 or FaDU cells transfected with EV, WT USP10, or C488A mutant, individually. **E** Competition-based assay to measure effect of USP10 shRNA on growth of Cal-27 and SCC-15 cells (*n* = 3 per time point). USP10-KD cells were identified by coexpression of green fluorescent protein (GFP) (LMN vector). The percentage of GFP^+^ cells was tracked over 12 days and normalized to GFP percentage on day 2. **F** cDNA complementation assay to demonstrate on-target effects of USP10 shRNA (*n* = 3 per time point). USP10 (linked to GFP, MSCV-based vector) was expressed in Cal-27 and SCC-15 cells before the expression of USP10 shRNA (linked to mCherry, LMN vector). The percentage of double positive cells was tracked and normalized to day 2 values. **G** Representative tumor pictures (left) of subcutaneous xenograft tumors were obtained from nude mice. Tumor growth curve was generated and compared (right). **H** Representative IHC and IF images showing the levels of Ki-67, CD31, and TUNEL in tumors derived from mice in EV and USP10 groups, individually. Data represent the Mean ± SD of at least three independent experiments. *P < 0.05, **P < 0.01, and ***P < 0.001. Differences were tested using a unpaired Student’s *t* test (**C**–**F**), and the 2-way ANOVA followed by Tukey’s multiple comparisons test (**A**, **B**, **G**).

To elucidate the biological roles of USP10 in HNSCC, we thus performed a series of functional assays. RT-qPCR analysis was used to detect USP10 mRNA levels in 7 HNSCC cell lines, and we accordingly categorized them into two groups (USP10^high^: Cal-27, SCC-15, SCC-25; and USP10^low^: Cal-33, FaDU, HN4, and UMSCC47) (Fig. S1A). Then, we overexpressed USP10 in USP10^low^ cell lines (Cal-33, FaDU) via lentivirus-mediated infection technology (Fig. S1B). CRISPR/Cas9 technology was used to delete the USP10 in USP10^high^ cell lines (Cal-27, SCC-15) (Fig. S1C). USP10 depletion impaired cell growth rates and colony formation efficiency, whereas USP10 overexpression significantly enhanced HNSCC cell growth (Fig. 2A–C). Next, we generated the catalytic-inactive USP10 mutant (USP10-C488A) with a mutation at the core enzymatic domain [31]. Only wild-type USP10, but not the USP10-CA mutant, could remarkably restore the impaired cell growth in USP10-depleted cells (Fig. 2D and Fig. S1D). Consistently, the catalytically deficient mutant failed to promote the clonogenic ability of Cal-33 or FaDU cells (Fig. S1E). Moreover, depletion of USP10 in Cal-27 and SCC-15 cells induced a decreased percentage of EdU-positive cells; conversely, USP10 overexpression led to an opposite effect in Cal-33 and FaDU cells (Fig. S1F, G). We also utilized a competitive proliferation assay to evaluate the effect of USP10 ablation on the growth of Cal-27 cells. Cal-27 and SCC-15 cells were rapidly outcompeted by nontransduced cells during culturing (Fig. 2E, F). Conversely, the expression of a human USP10 cDNA not recognized by USP10 shRNA reversed the impeded growth caused by shRNA (Fig. 2F). In addition, we conducted the tumor xenograft assays to elucidate the in vivo roles of USP10 and found that USP10 overexpression remarkably enhanced tumor growth compared with those of tumors derived from the control cells (Fig. 2G and Fig. S1H). Mice-bearing tumors derived from USP10-overexpressing cells suffered from shorter overall survival (OS) than those bearing control tumors (Fig. S1I). Histopathology also showed that USP10-OE tumors showed a more aggressive phenotype, greater proliferation based on Ki-67 staining, angiogenesis marker (CD31), and lower rates of apoptosis (Fig. 2H). Therefore, these data implicated that USP10 may act as an oncogene that enhances HNSCC growth in vitro and in vivo.

### USP10 promotes HNSCC metastasis in vitro and in vivo

**Fig. 3:**
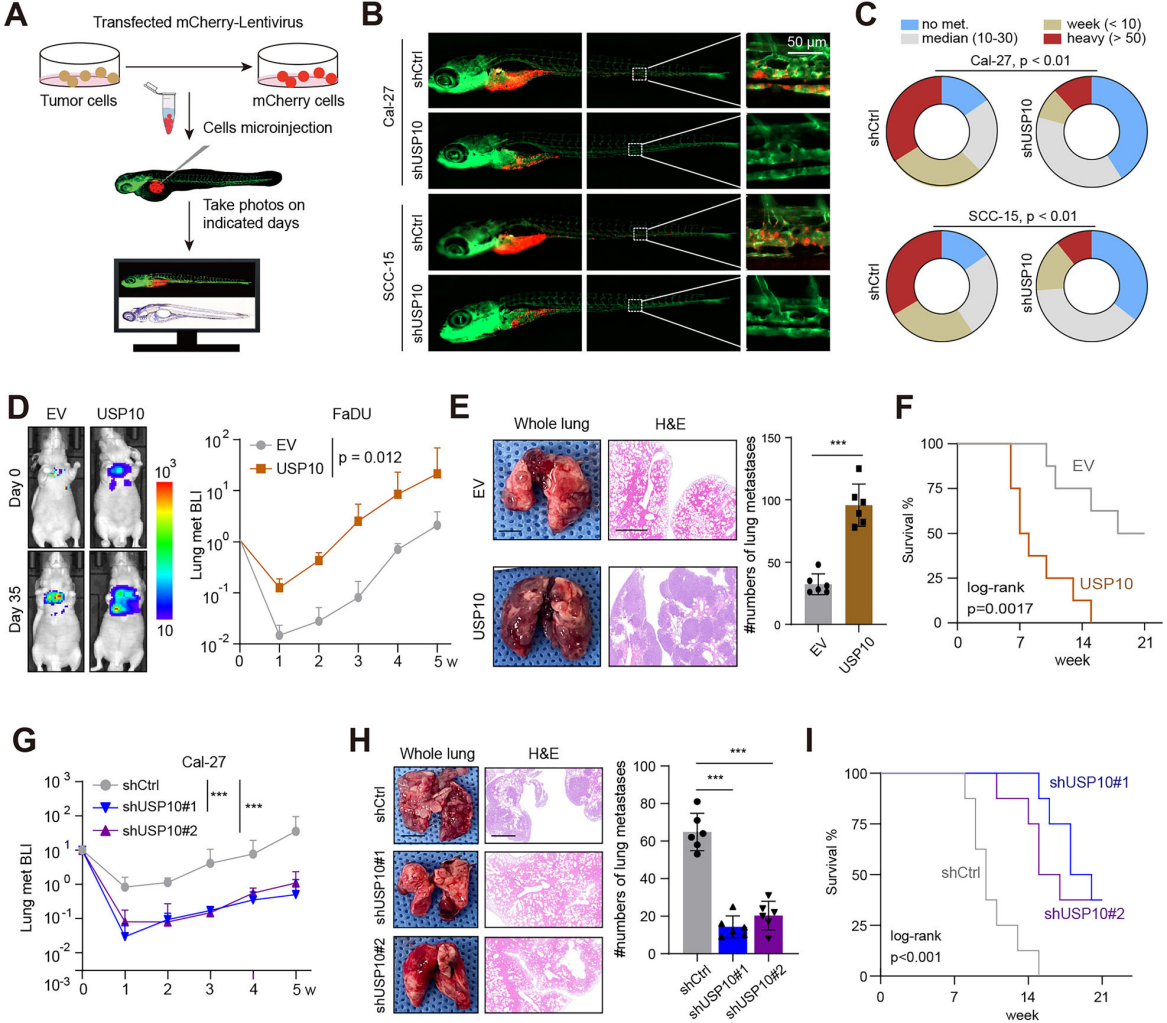
USP10 enhances HNSCC metastasis in vitro. **A** Workflow of HNSCC extravasation experiment in a zebrafish model. The blood vessels and cancer cells are fluorescently labeled in green and red, respectively. **B** Representative images of zebrafish from the control and the USP10-KD groups with zoom-in of invasive cells on the right panel. **C** Quantification of metastatic grades of HNSCC cells in tails of zebrafish. **D–F** The tail vein injection of FaDU cells with USP10 overexpression for lung colonization analysis. Shown are bioluminescent imaging (BLI) quantification and representative images (**D**), pulmonary surface nodules (**E**) (*n* = 5 mice per group), and animal Kaplan–Meier survival analysis (**F**). **G–I** The tail vein injection of Cal-7 cells with USP10 depletion for lung colonization analysis. Shown are BLI quantification (**G**), pulmonary surface nodules (**H**) (*n* = 5 mice per group), and animal Kaplan–Meier survival analysis (**I**). Data represent the Mean ± SD of at least three independent experiments. *P < 0.05, **P < 0.01, and ***P < 0.001. Differences were tested using an un-paired Student’s *t* test (**E**, **H**), the 2-way ANOVA followed by Tukey’s multiple comparisons test (**D**, **G**), and the log-rank test (**F**, **I**).

To characterize the roles of USP10 in HNSCC metastasis, we performed the migration and invasion assays in vitro. We first found that USP10 knockdown remarkably inhibited HNSCC cell migration and invasion (Fig. S2A, B). Wound-healing assay also revealed that Cal-27 cells with USP10 knockdown notably showed impaired cell migration relative to control cells, whereas USP10 overexpression promoted the cell migration (Fig. S2C, D). In addition, we detected the effects of USP10 on EMT- and metastasis-associated signature via qRT-PCR or western blot. Notably, N-cadherin, vimentin, and MTDH in USP10-OE cells were all reduced, while E-cadherin was significantly elevated (Fig. S2E). As is well known, zebrafish is an attractive animal model that is widely utilized in cancer research. We therefore overexpressed USP10 in mCherry-expressing FaDU cells and injected the circulation of zebrafish embryos (Fig. 3A), as previously instructed in our team [32]. After 6 days, we observed that USP10 overexpression both significantly promoted FaDU or Cal-33 cells extravasate into zebrafish tail fin and formed clusters (Fig. 3B, C). Furthermore, we generated a mouse xenograft model in which we intravenously injected FaDU cells stably expressing firefly luciferase, into BALB/c athymic nude mice. Noticeably, bioluminescent imaging (BLI) analyses revealed that USP10 overexpression in FaDU remarkably exacerbated lung-metastatic burden after intravenous inoculation of the cancer cells into mice (Fig. 3D, E, and Fig. S2F) and accordingly shortened animal survival (Fig. 3F, and Fig. S2G). In contrast, USP10 knockdown by short hairpin RNAs in Cal-27 notably alleviated metastatic burden and reduced lung colonization of the mice (Fig. 3G–I). These findings demonstrated a pro-metastatic role of USP10 in HNSCC.

### USP10 deubiquitinates BAZ1A to stabilize its proteins

**Fig. 4:**
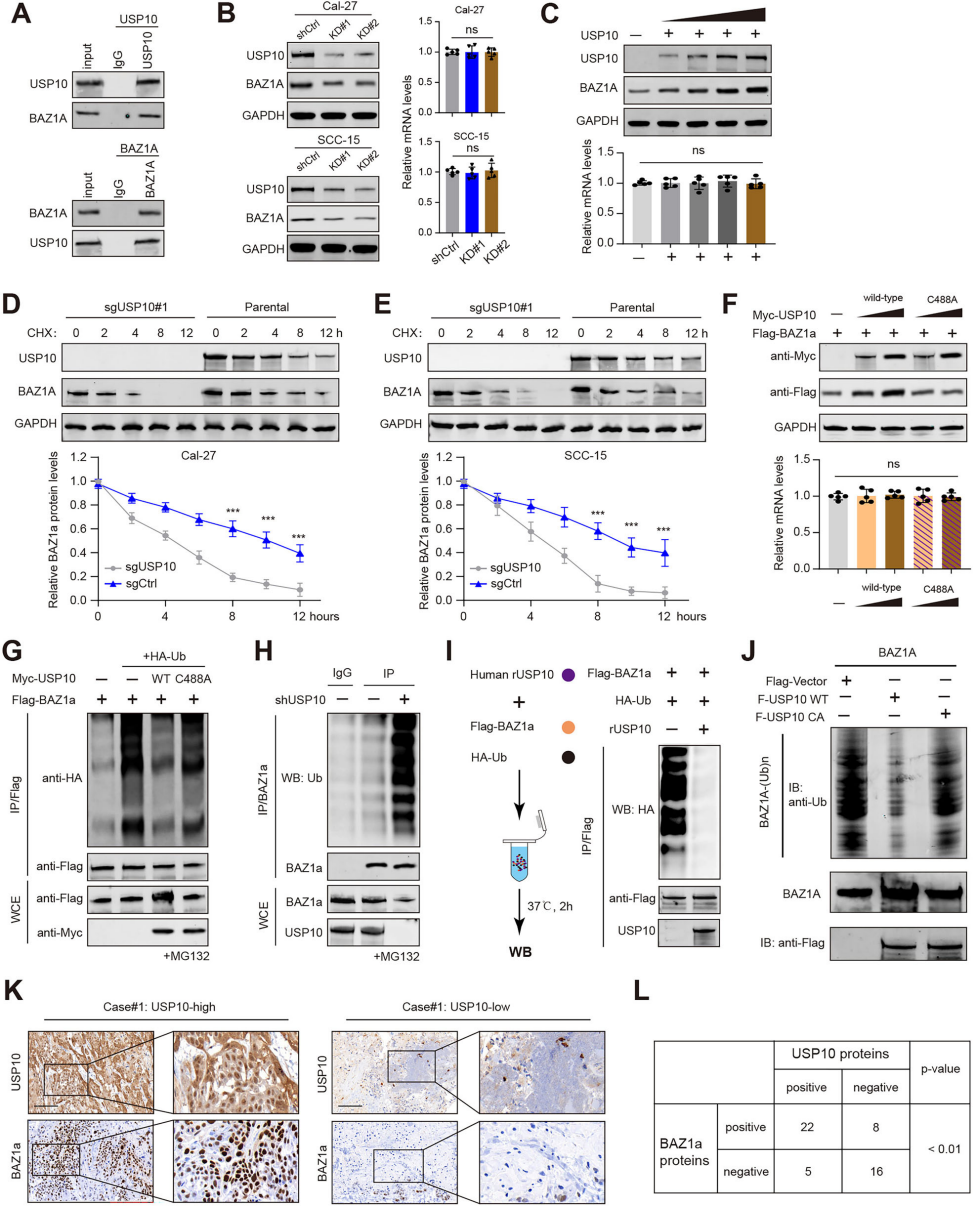
USP10 interacts with, deubiquitinates, and stabilizes BAZ1A. **A** Co-IP and western blotting analysis showing the endogenous interactions between USP10 and BAZ1A. **B** Western blotting and RT-qPCR analysis showing the BAZ1A expressions in indicated cells with or without USP10-KD. **C** Western blotting and RT-qPCR analysis showing the BAZ1A expressions in FaDU cells transfected with increased doses of Myc-USP10 plasmids. **D**, **E** Western blot of BAZ1A proteins in WCLs of Cal-27 (left) or SCC-15 (right) cells with or without USP10 depletion for 12 h and then treated with CHX (50 μg/ml) and harvested at different time points. At each time point, the intensity of BAZ1A was normalized to the intensity of GAPDH and then to the value at 0 h. Data are shown as means ± SD (*n* = 3). **F** Western blotting and RT-qPCR analysis showing the BAZ1A expressions in FaDU cells transfected with increasing doses of WT or C488A USP10 plasmids, individually. **G** In vivo ubiquitination assay was performed in cells transfected with Myc-USP10 (or C488A mutant), Flag-BAZ1A, or HA-ub. The cells were treated with 20 μM MG132 for 8 h, and western blotting showed the results. **H** In vivo ubiquitination assays showing the ubiquitination levels of BAZ1A in Cal-27 cells with or without USP10-KD. **I** The in vitro deubiquitination assay mode was illustrated (left). Deubiquitination of BAZ1A in vitro by recombinant USP10. HEK293FT cells transfected with BAZ1A and Ubiquitin constructs were treated with MG132 (10 μmol/L) for 8 h before harvest. **J** In vitro deubiquitination assay was performed. Ubiquitinated GFP-BAZ1A protein was treated with USP10 WT or USP10 CA. Reaction mixes were analyzed by western blotting. **K, L** Immunohistochemistry (IHC) images (**K**) showing USP10 and BAZ1A expressions in HNSCC specimens. Chi-square test to determine the correlations between USP10 and BAZ1A (**L**) in samples. Data represent the Mean ± SD of at least three independent experiments. *P < 0.05, **P < 0.01, and ***P < 0.001. Differences were tested using an un-paired Student’s *t* test (**B**, **C**, **F**), the 2-way ANOVA followed by Tukey’s multiple comparisons test (**D**, **E**).

To further clarify the molecular mechanism underlying the effect of USP10 on HNSCC progression, mass spectrometry and immunoprecipitation (IP) was utilized to screen and identify USP10-interacting proteins (Fig. S3A). Silver staining assay revealed that the USP10 immunoprecipitated group was observed with several specific bands of proteins compared to the IgG group. A list of peptides were identified in the complex, like DDX1, G3BP1, HDAC6, and BAZ1A (Fig. S3A). Given that epigenetic disorders contribute to HNSCC progression and BAZ1A is less studied, we thus selected BAZ1A for further investigations. Next, co-immunoprecipitation assays were conducted to show the physical binding between USP10 and BAZ1A (Fig. 4A). The immunoblotting assays detected decreased BAZ1A protein levels in USP10-depleted cells, whereas RT-qPCR analysis suggested that the BAZ1A mRNA levels did not alter (Fig. 4B). We further transfected the FaDU cells with increased doses of Myc-USP10 plasmids, and we found the BAZ1A proteins accumulated in a dose-dependent manner (Fig. 4C). Consistently, USP10 overexpression could not increase BAZ1A mRNA levels (Fig. 4C). Cycloheximide (CHX) assays verified that USP10 knockout significantly shortened the half-life of the BAZ1A proteins in Cal-27 and SCC-25 cells (Fig. 4D, E). However, only wild-type USP10, but not the CA mutant, could noticeably elevate BAZ1A proteins (Fig. 4F). Next, we intended to discover whether USP10 could efficiently remove the polyubiquitination of BAZ1A. As indicated in Fig. 4G, overexpression of wild-type USP10, but not of USP10-CA mutant, remarkably decreased the ubiquitination level of BAZ1A. Conversely, USP10 knockdown substantially elevated the level of ubiquitinated BAZ1A (Fig. 4H). Last of all, we conducted the in vitro deubiquitination assay via bacterial-expressed USP10. The ubiquitinated BAZ1A proteins were purified from the 293 T cells expressing Flag-BAZ1A and HA-Ub, and we incubated them with recombinant USP10 in a cell-free system (Fig. 4I). As expected, the purified USP10 could significantly deubiquitinate BAZ1A in vitro (Fig. 4I). To further confirm the findings, an in vitro deubiquitylation assay was conducted. Consistently, the wild-type USP10, but not the CA mutant, was effective in removing the Ub chains on BAZ1A in vitro (Fig. 4J). We further analyzed the expression of USP10 and BAZ1A in resected HNSCC samples. The immunohistochemistry analysis revealed that the expressions of USP10 were positively correlated with BAZ1A protein (*P* < 0.01; Fig. 4K, L). Together, we confirmed that USP10 interacts with BAZ1A to stabilize its proteins via the deubiquitination process.

### USP10 relied on BAZ1A to drive HNSCC stemness capacity and progression

**Fig. 5:**
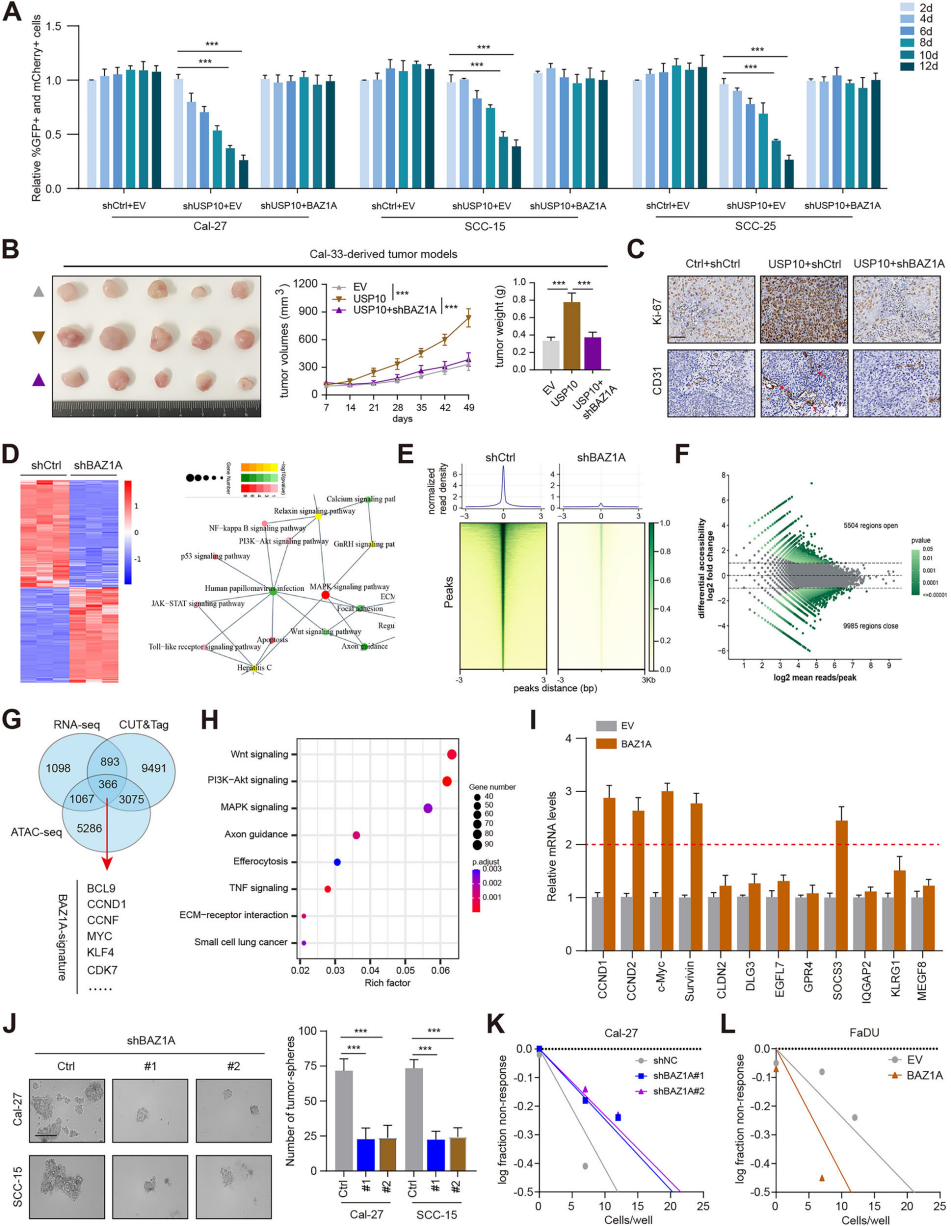
USP10 regulates HNSCC stemness and progression via positively regulating BAZ1A. **A** cDNA complementation assay to demonstrate on-target effects of BAZ1A shRNA (*n* = 3 per time point). USP10 (linked to GFP, MSCV-based vector) was expressed in indicated cells before expression of BAZ1A shRNA (linked to mCherry, LMN vector). The percentage of double-positive cells was tracked and normalized to day 2 values. **B** Representative images of subcutaneous xenograft tumors were obtained from nude mice in EV, USP10, and USP10+shBAZ1A groups. Tumor size and average weight were observed. **C** The IHC images showing the markers of Ki-67, and CD31 in indicated tumors. **D** Clustering heatmap (left) showing the differentially expressed genes in Ctrl and BAZ1A-KD cells. KEGG analysis (right) showing the enriched crosstalk by DEGs. **E** CUT&Tag-sequencing summary plot of BAZ1A-binding intensities across BAZ1A peaks in Cal-27 cells with versus without BAZ1A knockdown. **F** Identification of differential ATAC-seq signals showing the profiles of OCRs across the indicated genomic peaks in Cal-27 cells with versus without BAZ1A knockdown. **G** Venn diagram showing the intersected genes by overlapping DEGs, CUT&Tag-seq, and ATAC-seq data. **H** Dotplot showing the enriched pathways by BAZ1A-signature in (**G**). **I** RT-qPCR analysis showing the mRNA levels of CSC-related genes in EV and BAZ1A-overexpressing cells. **J** Representative graphs and quantification of the in vitro sphere-formation assay of BAZ1A knockdown HNSCC cells and control cells (*n* = 6). Scale bar=200 μm. **K** In vitro limiting dilution assay of BAZ1A-KD and control Cal-27 cells. A well not containing spheres (diameter ≥ 50 μm) was defined as a non-response (*n* = 12). **L** In vitro limiting dilution assay of control and BAZ1A-OE FaDU cells. Data represent the Mean ± SD of at least three independent experiments. *P < 0.05, **P < 0.01, and ***P < 0.001. Differences were tested using an un-paired Student’s *t* test (**A**, **B**, **I**, **J**), the 2-way ANOVA followed by Tukey’s multiple comparisons test (**B**).

To further investigate the role of BAZ1A in HNSCC, we extracted the expression data of BAZ1A from the TCGA-HNSC dataset and found BAZ1A was highly expressed in tumor samples than normal samples (Fig. S3B). We then designed two distinct shRNAs to delete BAZ1A and validated the knockdown efficiency (Fig. S3C). USP10 overexpression indeed enhanced the colony formation and migration abilities, which could be largely impaired by BAZ1A knockdown (Fig. S3D). Using a competitive proliferation assay, we evaluated the effect of BAZ1A restoration on the growth of USP10-depleted HNSC cells. The USP10-depleted Cal-27, SCC-15, and SCC-25 cells were rapidly outcompeted by those with BAZ1A restoration during culturing (Fig. 5A). To further figure out the in vivo functional relationships between USP10 and BAZ1A, we generated the xenograft mice model using stable USP10-overexpressing cells with or without BAZ1A knockdown. As expected, we found that USP10 overexpression remarkably enhanced in vivo tumor growth, which could be inhibited by BAZ1A knockdown (Fig. 5B). Correspondingly, IHC results revealed the elevated expressions of Ki-67 (a biomarker of proliferation), and CD31 (a marker of aginogenesis), in the tumor sections of the USP10-overexpressing group compared with that in the control group (Fig. 5C). BAZ1A knockdown could notably inhibit the expression levels of Ki-67 or CD31 in tumors derived from USP10-overexpressing cells (Fig. 5C). Therefore, we concluded that USP10 promoted HNSCC progression through the upregulation of BAZ1A expression.

To explore how BAZ1A helps maintain the transcriptional program in USP10-overexpressing HNSC, we reanalyzed the RNA-sequencing (RNA-seq) data to identify 3426 differentially expressed genes (DEGs) in response to BAZ1A depletion (Fig. S3E). These genes were mainly enriched in cancer-related biological items, including PI3K-AKT signaling, Wnt signaling, MAPK signaling, as well as JAK-STAT signaling (Fig. 5D). We further queried the CUT&Tag-sequencing data to confirm the epigenetic role of BAZ1A (Fig. 5E). After excluding false positives with BAZ1A-knockdown cells and filtering using a twofold increase criterion, we identified 15218 peaks associated with 13825 genes with a *P* value at <10^−5^ in Cal-27 cells (Fig. 5E). We further conducted an assay for transposase-accessible chromatin sequencing (ATAC-seq) to detect genome-wide changes in DNA accessibility between control and BAZ1A-knockdown cells. Dotplots of genome-wide ATAC-seq revealed that BAZ1A ablation caused at least a 40% decrease in open chromatin regions (OCRs), reducing the DNA accessibility at 9985 regions (Fig. 5F). To obtain the putative targets of BAZ1A in Cal-27 cells, we aligned the BAZ1A CUT&Tag peaks with the changes in OCRs and RNA-seq; 366 genes were therefore overlapped and defined as the BAZ1A-signature (Fig. 5G). KEGG analysis revealed that 366 genes were mainly enriched in the Wnt signaling pathway (Fig. 5H). RT-qPCR analysis further confirmed that several stemness-related targets were elevated in BAZ1A-overexpressing cells, including CCND1, CCND2, Survivin, and SOCS3 (Fig. 5I). However, the mRNA levels of the above targets were comparable in cells transfected with EV and BAZ1A-δBrd mutants (Fig, S3F). We then conducted the bioinformatic analysis to confirm the associations between BAZ1A and tumor stemness in HNSC samples. The one-class logistic regression (OCLR) machine-learning algorithm has been commonly utilized to decipher cancer stem-like indices (mRNAsi) in patients [33]. The TCGA-HNSCC patients were thus grouped into high- or low-stemness populations based on the mRNAsi value, and the BAZ1A-signature was preferentially enriched in patients from the high-stemness group (Fig. S3G, H). Furthermore, K-means clustering of samples from HNSCC patients based on the presence or absence of the BAZ1A-signature separated the cohort into two groups with substantial differences in survival (Fig. S3I). We further conducted several assays to test the relationships between BAZ1A and stemness in HNSCC. Interestingly, a decrease in sphere numbers and sizes as well as a markedly reduced stem cell frequency were observed in BAZ1A-KD Cal-27 and SCC-15 cells compared with the corresponding control cells (Fig. 5J, K). Conversely, BAZ1A overexpression could enhance the stemness of FaDU cells (Fig. 5L). Lastly, we also confirmed that USP10 relied on BAZ1A to enhance HNSC stemness in vitro (Fig. S3J). USP10 overexpression could activate HNSCC-related stemness markers, including CD44, and CD133 (Fig. S3K). In contrast, USP10 knockdown could reduce the levels of these stemness markers (Fig. S3L). Collectively, our data suggested that USP10 depended on BAZ1A to potentiate HNSCC self-renewal capacity and tumor progression.

### BAZ1A complexes with SOX2 to activate stemness-related signature via epigenetic remodeling

**Fig. 6:**
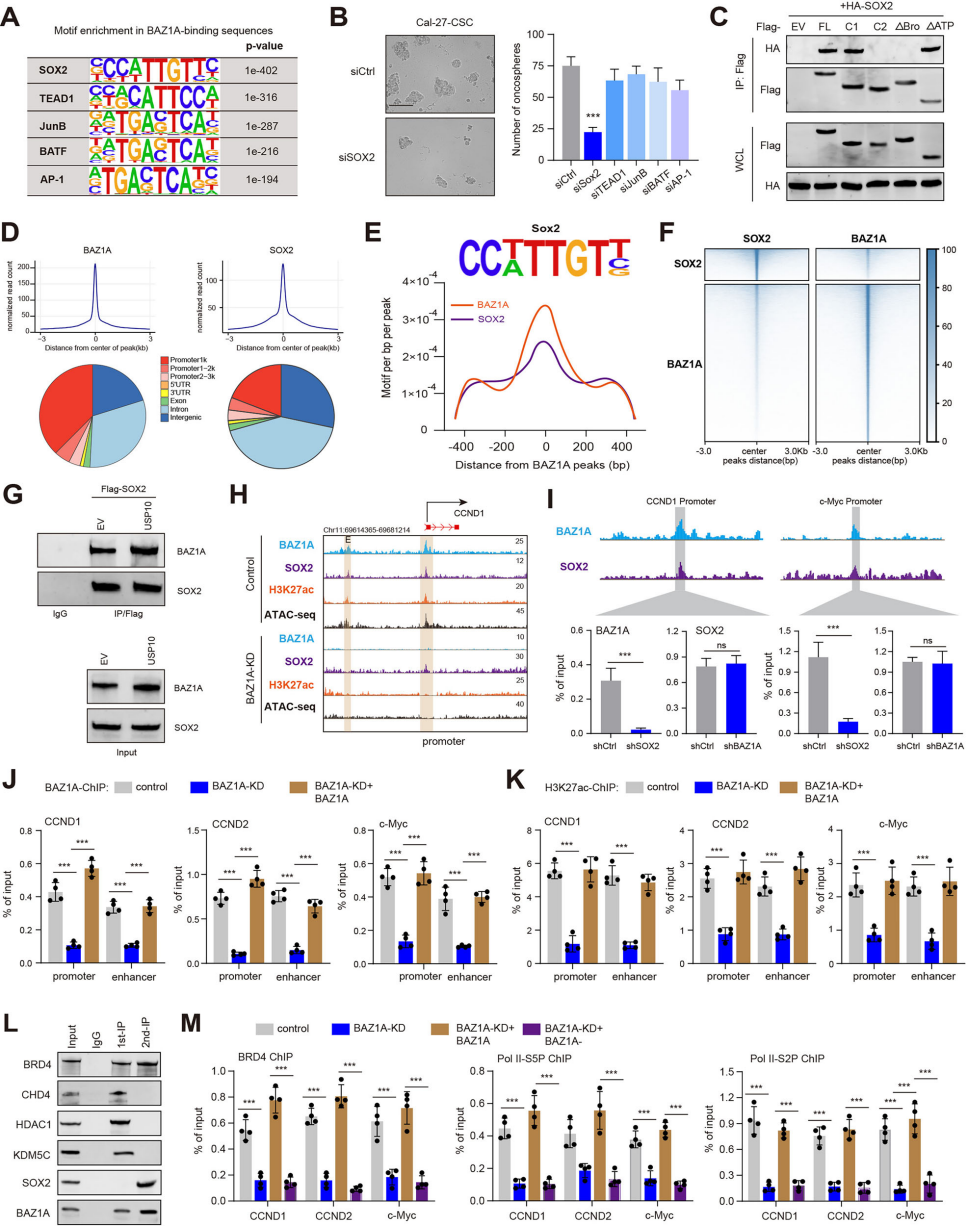
BAZ1A interacts with SOX2 to activate the expressions of CSC-related genes. **A** Enrichment of known recognition sequences (motifs) in BAZ1A-binding peaks. **B** Tumorsphere formation assays in control and Cal-27 cells transfected with indicated specific siRNAs. **C** IB analysis of immunoprecipitates of 293T cells transfected with the indicated plasmids. **D** Metagene analysis of the genomic distribution of BAZ1A (left) and SOX2 (right) in Cal-27 cells. TSS, transcription start site. **E** Motif density analysis of BAZ1A-SOX2 CUT&Tag-seq peaks. **F** Co-occupancy analysis of BAZ1A and SOX2 CUT&Tag-seq peaks. **G** Co-IP analysis showing the altered BAZ1A-SOX2 interactions in cells transfected with or without USP10 plasmids. **H** Gene tracks of CUT&Tag-seq signal for BAZ1A, SOX2, and H3K27ac at the CCND1 locus. The x-axis: Genomic position of promoter regions (P). The *y*-axis: CUT&Tag-seq signal (reads per million per base pair). **I** ChIP-qPCR analyses of BAZ1A or SOX2 on the indicated sites in Cal-27 cells treated with versus without shBAZ1A or shSOX2 (*n* = 3). **J, K** ChIP-qPCR analyses of BAZ1A (**J**) or H3K27ac (**K**) binding on the promoters or enhancers of genes in BAZ1A-KD cells with or without BAZ1A restoration. **L** IP-re-IP assays in Cal-27 cells stably transfected with the indicated plasmids. **M** ChIP-qPCR analysis of BRD4 (left), Pol II-S5P and S2P (right) in the promoter regions of the indicated genes in WT and BAZ1A-KD Cal-27 cells stably restoring WT BAZ1A as indicated (*n* = 4). Data represent the Mean ± SD of at least three independent experiments. *P < 0.05, **P < 0.01, and ***P < 0.001. Differences were tested using an unpaired Student’s *t* test (**B**, **I**, **J**, **K**, **M**).

To further clarify the mechanism underlying the transcription of BAZ1A signature genes, we enriched transcription factor binding motifs in BAZ1A-bound regions. Among these BAZ1A-binding sites, motifs recognized by SRY-box transcription factor 2 (SOX2), TEA domain transcription factor 1 (TEAD1), JunB proto-oncogene, AP-1 transcription factor subunit (JUNB), and basic leucine zipper ATF-like transcription factor (BATF) were notably enriched (Fig. 6A). However, knocking down of SOX2, but not the other TFs, could efficiently inhibit HNSCC cell’s renewal abilities (Fig. 6B). Consistently, SOX2 was associated with FL BAZ1A but not with BAZ1A-δBromodomain (Fig. 6C, and Figure S4A). We further showed that the Bromodomain-truncated mutant (δBro) failed to stimulate SOX2-dependent transcription (Fig. S4B), indicating that the interaction-dependent activation of the stemness pathway. Then, we performed CUT&Tag-sequencing with anti-SOX2 in Cal-27 cells to confirm whether BAZ1A colocalizes with SOX2 at the binding sites. As implicated, BAZ1A was highly enriched near the transcription start sites, similar to SOX2 (Fig. 6D). The motif analysis of BAZ1A-SOX2-binding peaks further revealed marked enrichment of the Sox2 motif (Fig. 6E). The metagene analysis confirmed strong colocalization of SOX2 and BAZ1A (Fig. 6F). We further conducted IP experiments in 293 T cells and observed that the interaction between BAZ1A and SOX2 was increased following USP10 overexpression (Fig. 6G). To determine whether BAZ1A binds to the SOX2-directed sites through modified histones, we studied the occupancy of H3K27ac on the promoters of target genes. CUT&Tag data suggested that H3K27ac peaks were markedly overlapped with BAZ1A peaks and co-occupied at the promoters of genes driven by SOX2, like *CCND1*, and *c-Myc* (Fig. 6H, and Fig. S4C). However, the H3K27ac enrichment was markedly impaired by BAZ1A knockdown, along with decreased chromatin accessibility (Fig. 6H, and Fig. S4C). Intriguingly, SOX2 peaks were not altered by BAZ1A depletion (Fig. 6H, and Fig. S4C). Therefore, we questioned whether BAZ1A and SOX2 bound to promoters sequentially or simultaneously. ChIP-qPCR analysis at *CCND1* and *c-Myc* promoter sites showed that BAZ1A occupancy was ablated after SOX2 knockdown, whereas BAZ1A knockdown did not impair SOX2 binding, implicating that SOX2 directs BAZ1A to promoters of targets (Fig. 6I). ChIP-qPCR analysis further confirmed that BAZ1A depletion impaired BAZ1A-, and H3K27ac-binding to promoters of targets, which could be significantly restored by BAZ1A ectopic expression (Fig. 6J, K). As reported, dCas9-KRAB technology with specific sgRNAs targeting the enhancers of genes was used to induce enhancer inefficiency via constructing a heterochromatin-forming complex [32]. We confirmed that enhancers inactivation by sgRNAs significantly abolished enhancer-promoter contacts, and BAZ1A-binding to chromatin regions, leading to decreased CCND1/c-Myc expressions (Fig. S4D). Next, we conducted a stepwise immunoprecipitation (IP) assay to characterize BRD4/SOX2-associated factors. The Cal-27 cells were transfected with Flag-BAZ1A and Myc-SOX2. The first round of IP was performed by an anti-FLAG antibody, while the second round (re-IP) used an anti-Myc antibody. We observed that BAZ1A complexes with NuRD complex components, like CHD4, HDAC1, and KDM5C (Fig. 6L). However, only BRD4 could be enriched by sequential BAZ1A/SOX2 IP procedures (Fig. 6L). BAZ1A knockdown could efficiently prevent the recruitment of BRD4 to the promoter regions of *CCND1, c-Myc*, and *CCND2*. Furthermore, ChIP-qPCR analysis revealed that both Ser5- and Ser2-phosphorylated RNA Pol II CTDs were inhibited (Fig. 6M). These data show that BAZ1A/SOX2 associates with BRD4 to stimulate the stemness-related signature.

### Targeting BAZ1A attenuates HNSCC stemness and enhances chemotherapy sensitivity

**Fig. 7:**
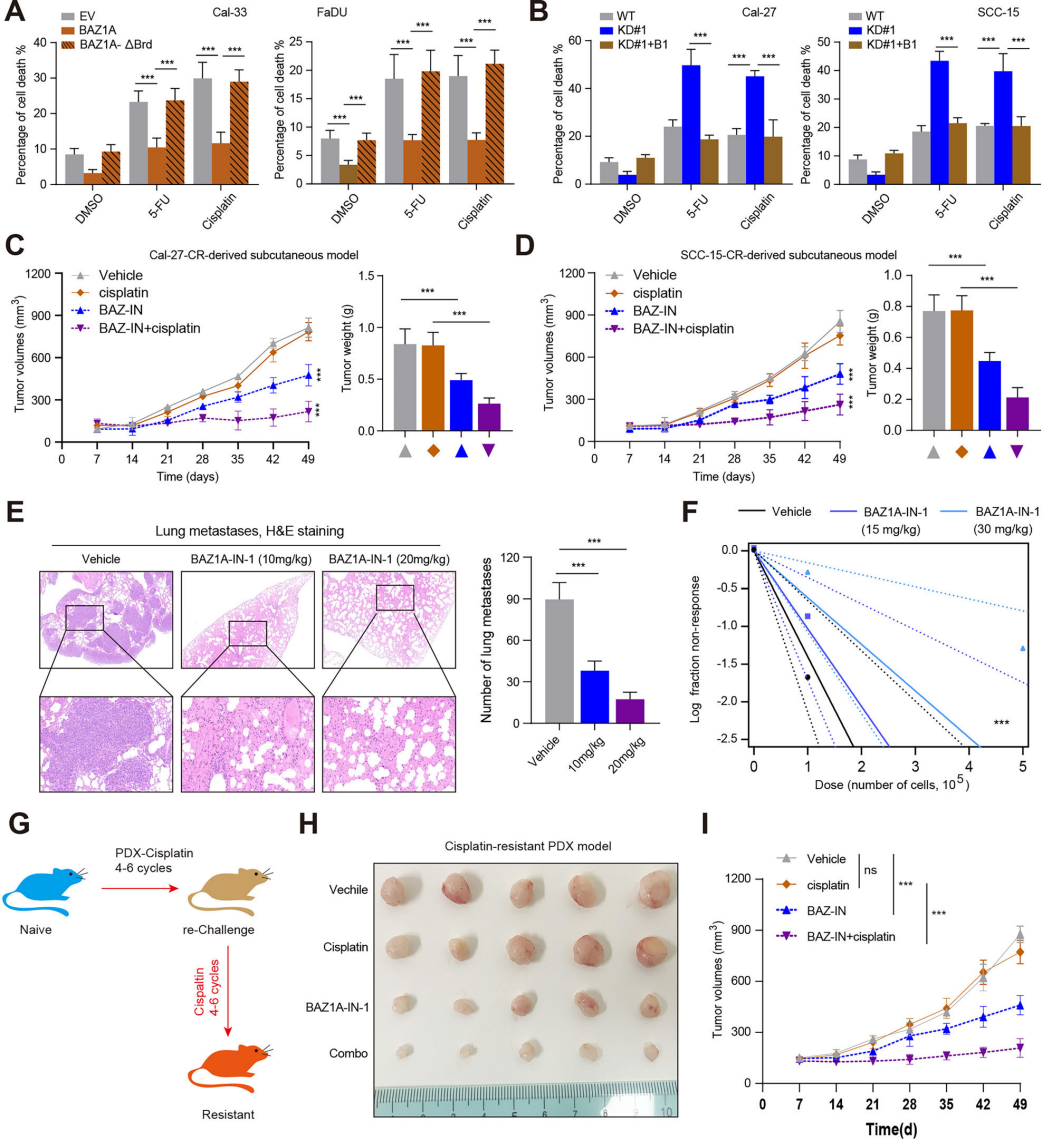
USP10-BAZ1A axis confers chemotherapy resistance in HNSCC. **A** The cell death analysis (PI staining) of Cal-33 (left) or FaDU (right) cells stably expressing EV, BAZ1A, or BAZ1A-ΔBro treated with DMSO, 5-FU, or cisplatin. **B** The cell death analysis (PI staining) of BAZ1A-KD Cal-27 (left) or SCC-15 (right) cells with or without BAZ1A restoration treated with DMSO, 5-FU, or cisplatin. **C, D** Cal-27-CR- (**C**) or SCC-15-CR-derived (**D**) subcutaneous models showing the in vivo efficacy of cisplatin, or BAZ1A-IN-1 in suppressing tumors. **E** Representative H&E staining (scale bar: 100 μm) and quantification of metastatic lung nodules at day 60 after the tail vein injection of Cal-27 cells in mice treated with or without BAZ1A-IN-1 treatment. **F** In vivo limiting dilution assay showing the estimated frequency of CSCs among Cal-27 cells with or without BAZ1A-IN-1 treatment. Response: mice developed subcutaneous tumors (*n* = 5 mice per group). **G** Schematic graphs of acquired cisplatin resistance HNSCC-PDX model generation. **H, I** Representative tumor image and quantification curves showing the in vivo efficacy of cisplatin, BAZ1A-IN-1, and Combo in suppressing the growth of HNSCC-PDX-CR. Data represent the Mean ± SD of at least three independent experiments. *P < 0.05, **P < 0.01, and ***P < 0.001. Differences were tested using an unpaired Student’s *t* test (**A**, **B**, **E**), and the 2-way ANOVA followed by Tukey’s multiple comparisons test (**C**, **D**, **I**).

**Fig. 8:**
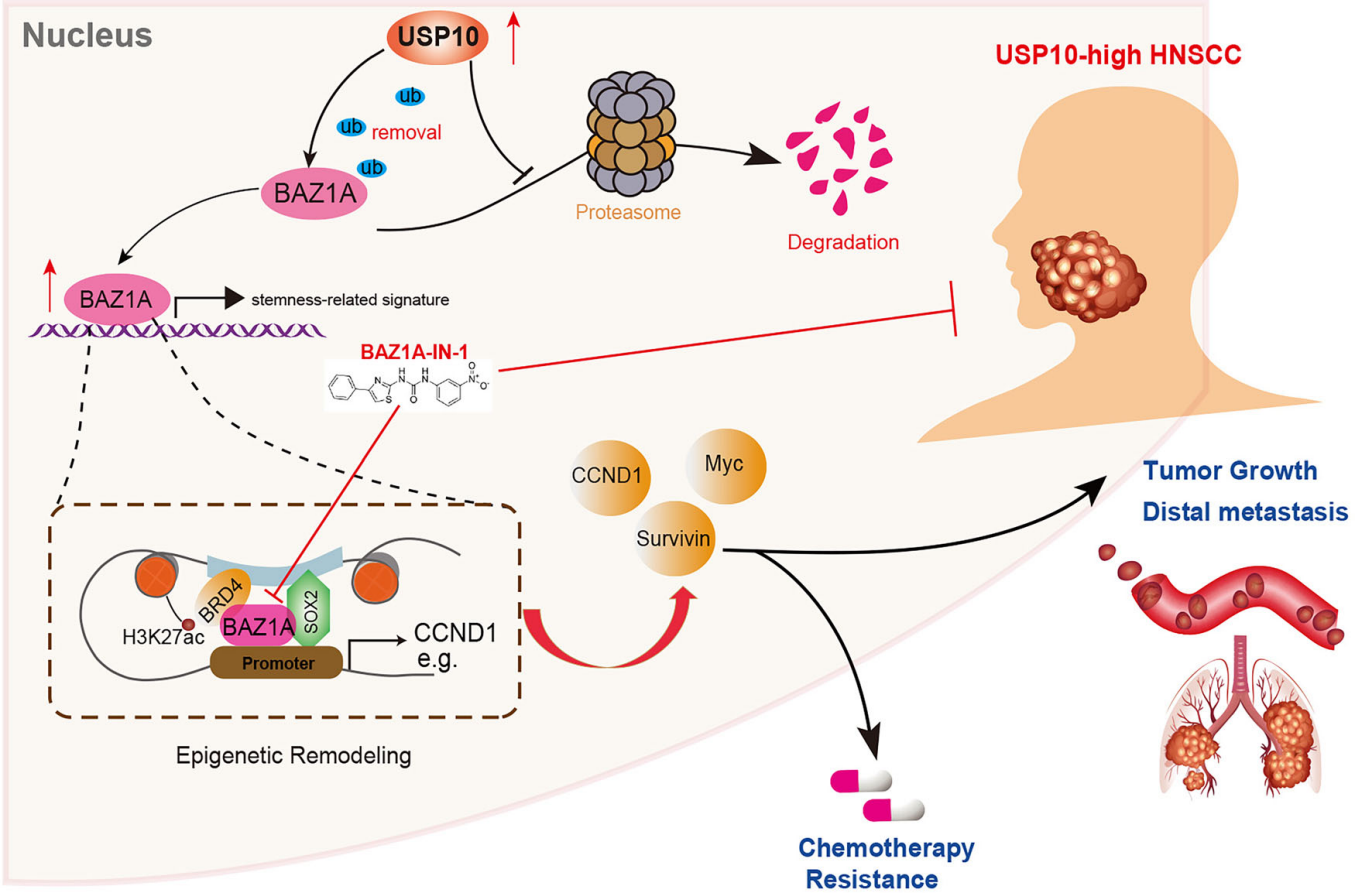
Graphical working model of USP10-BAZ1A-SOX2 regulating axis in this study.

Considering that enhanced cancer stem cell-like features contribute to chemoresistance, we, therefore, intended to confirm the associations between USP10/BAZ1A axis and chemotherapy resistance in HNSCC. First of all, we observed that WT BAZ1A, but not the BAZ1A-δBro mutant, could protect cancer cells from 5-FU, cisplatin-induced cell death as measured by propidium iodide staining (Fig. 7A). In contrast, BAZ1A knockdown could render HNSCC cells sensitive to 5-FU, or cisplatin treatment (Fig. 7B). In addition, we generated two cisplatin-resistant HNSCC cell lines, named Cal-27-CR, and SCC-15-CR (Fig. S5A). Then, we constructed the subcutaneous tumor model using the Cal-27-CR, or SCC-15-CR cells. As expected, we found cisplatin treatment could induce a mild effect on tumors derived from Cal-27-CR, or SCC-15-CR cells (Fig. 7C, D). We further utilized the specific BAZ1A inhibitor, BAZ1A-IN-1, to treat the HNSCC model in mice. Notably, prolonged BAZ1A-IN-1 treatments showed no evident toxicity in immuno-deficient mice, with no impact on body weight (Fig. S5B), and complete blood counts (Fig. S5C). Then, we established the tail vein injection model with Cal-27 cells, and the mice were treated with BAZ1A-IN-1 (20 mg/kg per day). We confirmed that BAZ1A-IN-1-treated mice developed fewer lung metastatic nodules, as evidenced by histologic examination (Fig. 7E). As mentioned above, USP10/BAZ1A maintained self-renewal ability in vitro; therefore, we queried whether a similar effect would exist in vivo. Notably, the frequency of tumorigenic HNSCC cells was significantly decreased among the BAZ1A-IN-1-treated Cal-27 cells (Fig. 7F). Lastly, to simulate acquired cisplatin resistance in vivo, we utilized an analogous approach of repeated chemotherapy cycles in human PDX models of HNSCC to select populations of tumors that were resistant to cisplatin (Fig. 7G). The expression of CSC-associated genes, including MYC, SOX2, OCT4, NANOG, CCND1, and CCND2 were analyzed in two paired PDX models with or without cisplatin resistance (Fig. S5D). The BAZ1A-IN-1 treatment could significantly suppress the growth of PDXs, compared with vehicle, or cisplatin administration (Fig. 7H). Of note, the combined strategy of BAZ1A-IN-1 and cisplatin could lead to more inhibitory effects on tumor growth than either one alone (Fig. 7H, I). The IHC analysis confirmed that BAZ1A-IN-1 could effectively down-regulate the CSC-related genes in vivo, like *c-Myc, CCND1*, and *EpCAM* (Fig. S5E). Taken together, these results suggested that BAZ1A inhibitor could suppress HNSCC growth, and metastasis, and strengthen cisplatin efficacy (Fig. 8).

## Discussion

Dysregulated ubiquitination modifications are associated with various types of cancer, including HNSCC [34]. Through bioinformatic analysis, we identified USP10 as a critical factor for HNSCC progression. High USP10 in HNSCC predicted poor prognosis of patients and is an independent hazard factor. Functional assays confirmed that USP10 could potentiate HNSCC cell proliferation and metastatic capacities in vitro and in vivo. USP10 physically interacts with BAZ1A and mediates the deubiquitination of BAZ1A proteins, leading to the stabilization of BAZ1A. BAZ1A complexes with SOX2 to activate stemness-associated genes via epigenetic remodeling, including CCND1, CCND2, c-Myc, and Survivin, e.g. Furthermore, BAZ1A recruits BRD4 to SOX2-binding promoters to sustain the chromatin accessibility and facilitate the H3K27ac enrichment. In addition, we found that the USP10-BAZ1A axis could enhance tumor stemness and HNSCC chemotherapy resistance. BAZ1A specific inhibitor, BAZ1A-IN-1, could effectively inhibit the in vivo growth of cisplatin-resistant tumors, and strengthen the cisplatin efficacy. Last of all, we generated cisplatin-resistant patient-derived xenografts (PDXs) and validated that BAZ1A-IN-1 could render PDX-CR sensitive to cisplatin administration.

USP10 is a significant member of the ubiquitin-specific protease family, extensively expressed in the cytoplasm and nucleus of nearly all cell types [35]. As a deubiquitinating enzyme, USP10 can reverse substrate ubiquitination by cleaving ubiquitin (Ub) molecules from the C-terminal of Ub-conjugated target proteins, thereby preserving intracellular protein homeostasis through Ub recycling [36]. Abnormal high expressions of USP10 were reported to be a pro-tumorigenic factor in multiple tumors [37, 38]. Previous studies revealed that USP10 participates in YAP-TEAD and p53 pathways. For instance, USP10 functions as a DUB of YAP/TAZ and drives the proliferation of hepatocellular carcinoma via stabilizing the YAP/TAZ complex [18]. Following DNA damage, USP10 is stabilized, and a portion of USP10 translocates to the nucleus to activate p53 [39]. ATM-mediated phosphorylation of USP10 contributes to the translocation and stabilization of USP10. Finally, USP10 cooperates with p65 to suppress tumor cell growth [35]. However, Chen Hu et al. found that USP10 could interact with, deubiquitinate, and stabilize oncogenic protein histone deacetylase 6 (HDAC6). Elevated USP10 levels correlate with poor overall survival in NSCLC with mutant p53 [40]. Therefore, USP10 may have dual roles in regulating p53 signaling. In this study, we uncovered the novel roles of USP10 in regulating HNSCC stemness. Particularly, USP10 elevates stemness-related genes via stabilizing BAZ1A proteins and depends on BAZ1A to enhance self-renewal abilities. USP10 further enhances HNSCC cisplatin resistance via BAZ1A/SOX2 epigenetic regulations. Therefore, it is of great importance to identify novel roles of USP10 in potentiating HNSCC progression and drug resistance.

Chromatin remodeling is a dynamic and regulated process that modulates DNA accessibility to various regulatory proteins, thus altering the transcription of adjacent genes [41]. Transcription typically necessitates ATP-dependent chromatin remodelers to modify histone-DNA interactions and to mobilize, evict, or replace nucleosomes [42]. BAZ1A (bromodomain adjacent to zinc finger domain, 1A), also known as ACF1, is an accessory, non-catalytic subunit of ACF (the ATP-dependent chromatin assembly factor) [43]. It is believed that BAZ1A controls the template specificity of the ATPase component SNF2H and improves nucleosome sliding efficiency [44]. BAZ1A has been documented to regulate the ATPase activity of the ACF complex and to be involved in gene transcription, DNA damage checkpoint mechanisms, and double-strand break repair [45]. BAZ1A can also play a role in neurodevelopment and spermatogenesis by modulating the expression of related genes [46]. Whether BAZ1A proteins have specific roles in tumorigenesis remains unclear. In our study, we found that BAZ1A mainly regulates Wnt and stemness-related signaling in HNSCC. BAZ1A could interact with SOX2 to drive the expressions of downstream targets. Intriguingly, only WT BAZ1A, but not the BAZ1A-δBromodomain, could drive stemness crosstalk. We explained that the Bromodomain may be the essential structure for the interactions between BAZ1A and SOX2. In addition, we uncovered that BAZ1A could recruit BRD4 to the BAZ1A-SOX2 co-occupied sequences, and we observed preferential H3K27ac enrichment at these sites. BRD4 is recognized for its interaction with positive transcription elongation factor b (P-TEFb) and modulates P-TEFb–mediated phosphorylation of RNA polymerase II at serine 2, thereby facilitating the release of stalled RNA polymerase II at the promoter [47]. Recent investigations indicate that RNA polymerase II pausing and release precisely regulate transcriptional activation [48]. As a result, the BAZ1A/BRD4 complex effectively corroborates the existing paradigm of SOX2 machinery activation and signifies a crucial molecular mechanism facilitating the release of stalled RNA polymerase II.

The study still has some limitations that deserve to be addressed. First of all, we may need to collect more HNSCC samples to define high-USP10 or BAZ1A samples. Secondly, we only discovered the functions of the bromodomain of BAZ1A in interacting with SOX2 and driving the stemness program. However, BAZ1A also has other essential domains, including DDT (423–486), WHIM1 (593–638), PHD (1150–1198), and ATP-related domains (22–122). Whether BAZ1A regulates chromatin assembly and remodeling via other domains is still unclear. Another limitation of our study is the inability to ascertain the contribution of the adaptive immune response to the anticancer effect of BAZ1A-IN-1, as athymic nude mice were utilized for human tumor xenograft experiments. Further research in immunocompetent hosts will be necessary to resolve this issue. Previous studies have already implicated the relationships between USP10 and DNA damage, and high USP10 could facilitate ESCC cells to obtain acquired radioresistance [49, 50]. Since we have uncovered the potential roles of USP10-BAZ1A axis in sustaining stemness process, we could further confirm whether targeting USP10-BAZ1A could sensitize HNSCC cells to radiotherapy. The synergistic effect of BAZ1A-IN-1 and radiotherapy should be thoroughly evaluated.

Together, our study revealed that USP10-BAZ1A acts as an oncogenic axis to promote HNSCC growth, metastasis, and cisplatin resistance by maintaining stemness features. BAZ1A coordinates with SOX2 to sustain expressions of CSC-related genes via epigenetic remodeling. Thus, targeting BAZ1A is an effective strategy to suppress USP10-high HNSCC and enhance cisplatin sensitivity.

## Materials and methods

### Cell culture

HNSCC cell lines Cal-27 (#CRL-2095), SCC-15 (#CRL-1623) and FaDU (#HTB-43) were purchased from the American Type Culture Collection (ATCC), and Cal-33 (#ACC-447) was obtained from Deutsche Sammlung von Mikroorganismen und Zellkulturen (DSMZ). HNSCC cell lines were cultured in Dulbecco’s Modified Eagle Medium (DMEM) supplemented with 10% fetal bovine serum (FBS) and penicillin/streptomycin solution. The cells were maintained in a humidified incubator at 37 °C with 5% CO_2_. The cell lines were recently validated by short tandem repeat (STR) profiling and assessed for mycoplasma contamination.

### Plasmids and siRNA

Lentiviral pLKO.1 plasmid vectors targeting USP10 and BAZ1A were constructed according to the protocol of pLKO.1-puro vector (Addgene) to establish individual stable cells. The following are the oligonucleotide sequences that were designed and listed: shUSP10#1: 5’-CCGGCCTATGTGGAAACTAAGTATTCTCGAGAATACTTAGTTTCCACATAGGTTTTTG-3’ (Forward), 5’-AATTCAAAAACCTATGTGGAAACTAAGTATTCTCGAGAATACTTAGTTTCCACATAGG-3’ (Reverse); shUSP10#2: 5’-CCGGGCTGTGGATAAACTACCTGATCTCGAGATCAGGTAGTTTATCCACAGCTTTTTG-3’ (Forward), 5’-AATTCAAAAAGCTGTGGATAAACTACCTGATCTCGAGATCAGGTAGTTTATCCACAGC-3’ (Reverse); shBAZ1A: 5’-CCGGCGACCGCATGTATAGACGATACTCGAGTATCGTCTATACATGCGGTCGTTTTTG-3’ (Forward), 5’-AATTCAAAAACGACCGCATGTATAGACGATACTCGAGTATCGTCTATACATGCGGTCG-3’ (Reverse). Transient gene knockdown were performed by transfecting siRNAs (60–100 nM) using Lipofectamine 2000 (Invitrogen) according to a standard procedure. The siRNAs targeting sequences are listed as follows: SOX2: 5’-TGGACAGTTACGCGCACATGA-3’; TEAD1: 5’-GCTCAAACACTTACCAGAGAA-3’; JunB: 5’-CCCATCAACATGGAAGACCAA-3’; BATF: 5’-GAGAAACAGAACGCGGCTCTA-3’.

### RNA extraction and real-time PCR

Total RNAs were isolated from cells or tissues utilizing TRIzol solution (Invitrogen). The quantity and quality of total RNAs were assessed with a NanoDrop 2000 spectrophotometer (Thermo). Then, total RNAs were reverse-transcribed into cDNA utilizing a Superscript RT kit (TOYOBO) with random primers, followed by qPCR amplification employing the SYBR Green PCR Master Mix Kit (TOYOBO). Gene expression alterations were standardized to the endogenous GAPDH level and quantified utilizing the 2^-ΔΔCT methodology. The subsequent list comprises the primers that were employed (5’- > 3’): USP10:

ATTGAGTTTGGTGTCGATGAAGT (Forward), GGAGCCATAGCTTGCTTCTTTAG (Reverse); BAZ1A: AGTTGTGCTGTGACGGGTAG(Forward), GTAAGCGCGAACGATGGGTAA (Reverse); SOX2: TACAGCATGTCCTACTCGCAG (Forward), GAGGAAGAGGTAACCACAGGG (Reverse); c-Myc: GTCAAGAGGCGAACACACAAC (Forward), TTGGACGGACAGGATGTATGC (Reverse); CCND1: GCTGTGCATCTACACCGACA (Forward), TTGAGCTTGTTCACCAGGAG (Reverse); OCT4: CTTGAATCCCGAATGGAAAGGG (Forward), GTGTATATCCCAGGGTGATCCTC (Reverse); NANOG: CCCCAGCCTTTACTCTTCCTA (Forward), CCAGGTTGAATTGTTCCAGGTC (Reverse).

### Cell viability assay and colony formation assays

The Cell Counting Kit-8 (CCK-8; Dojindo) assay assessed the viability of the RCC cells in accordance with the manufacturer’s guidelines. The cells were inoculated on a 96-well plate at a density of 2,000 cells per well. Following the treatments, the medium was incubated for 2 h at 37 °C with 10 μl of CCK-8 solution added. A microplate reader was used to measure absorbance at 450 nm, and the results were standardized to the control. Cell viability was determined based on the outcomes of three independent experiments. For colony formation assay, HNSCC cells were inoculated at a density of 1.5 × 10³ cells per well in 6-well plates, with three replicates. Following a two-week period, the colonies were rinsed, treated with 4% paraformaldehyde, and stained with 0.1% crystal violet to enable colony enumeration.

### Western blotting

Cells and tissues were lysed with RIPA solution supplemented with a protease inhibitor (MCE) and were subjected to SDS-PAGE. The proteins were then transferred to polyvinylidene difluoride (PVDF) membranes (Millipore). The membranes were blocked with 5% non-fat milk for 1 h and subsequently incubated with primary antibodies overnight at 4 °C. The following day, the membranes were incubated with secondary antibodies for 1 h at room temperature and visualized using an ECL chemiluminescence system (Santa Cruz). Following are the antibodies utilized for Western blotting: anti-USP10 (Abcam, ab72486), anti-BAZ1A (MilliporeSigma, HPA002730), anti-GAPDH (Cell Signaling Technology, #2118), anti-Myc (Cell Signaling Technology, #2278), anti-Flag (Cell Signaling Technology, #14793), anti-HA (MilliporeSigma, H6908), anti-BRD4 (Abcam, ab128874), anti-CHD4 (Abcam, ab72418), anti-HDAC1 (Abcam, ab7028), anti-KDM5C (Proteintech, 14426-1-AP) and anti-SOX2 (Abcam, ab97959). The uncropped images of blots were gathered and shown in Figure S6.

### Poly-ubiquitination detection assay

The poly-ubiquitination of BAZ1A was observed by co-transfecting cells with 2 µg of Myc-USP10 or Myc-tag, 0.5 µg of HA-Ub plasmids, and 0.5 µg of Flag-BAZ1A for a duration of 24 h. Protein extraction was performed 6 h post-treatment with 10 μM MG132. Pre-clearance was performed using 30 µL of protein A agarose (P2051, Beyotime) for a period of 2 h. Subsequent to this procedure, the extract was treated overnight with an anti-Flag antibody. The entire polyubiquitinated BAZ1A was identified using an anti-HA antibody by western blotting.

### In vitro ubiquitination assay

After being sown in 6 cm culture dishes for the entire night, the cells were cultured for 48 h with either 4 μM Tim-AIII or an equivalent volume of DMSO. After that, 10 μM MG-132 was added to stop the protein from breaking down for 4 h. Following incubation, the cells were collected and subjected to IP lysis solution (P0013J, Beyotime) to extract proteins. The antibody was then incubated with the cell lysates for a whole night at 4 °C. Subsequent to an additional incubation with protein A/G Sepharose beads (P2179, Beyotime) for 3 h, the beads were subjected to five washes with IP lysis solution before the addition of loading buffer for Western blotting.

### Immunoprecipitation assay and mass spectrometry

To validate the potential USP10-interacting proteins, co-transfected cells expressing Flag-, and HA-tagged USP10 were constructed. Immunoprecipitation was carried out using anti-DYKDDDDK beads and anti-HA beads to enrich USP10 proteins. After that, we performed SDS-PAGE and stained the gel using Coomassie brilliant blue solution. Subsequently, we gathered the protein bands and employed LC-MS/MS for analysis. The peptides were isolated using a Thermo Scientific EASY-nLC 1000 system. The extracted peptide was identified using a Q-Exactive mass spectrometer (Thermo Scientific). Mascot software was employed to analyze the raw data by conducting a search of the human UniProt database.

### Immunohistochemistry (IHC)

The study was performed in line with ethical guidelines of Declaration of Helsinki. We obtained the written informed consent from the HNSCC patients, and this study was in compliance with the ethical standards set by the Ethics Committee of Shanghai Ninth People’s Hospital. All participants in the study provided informed consent. Participants received a comprehensive elucidation of the study’s objectives, methodologies, and possible risks and advantages prior to the acquisition of consent. IHC was performed using an anti-USP10 antibody (1:50, #ab109219, Abcam). Each sample received a score based on the staining intensity and the ratio of stained cells. Three independent clinical pathologists evaluated the stained tissues.

### CUT&Tag, ATAC-seq, RNA-seq, and data analysis

Adaptor removal from the raw data obtained by CUT&Tag sequencing was executed using Trimmomatic (v0.39), retaining reads longer than 36 base pairs. The resultant clean data were aligned to the GRCm39 reference genome via bowtie2 (v2.5.1). PCR duplicates were removed by sambamba markdup. Reads per kilobase per million mapped reads (RPKM) for 100 base pair genomic bins were used to normalize read counts in subsequent analyses. MACS2 (v2.2.9.1) was used to identify peaks with the following parameters: -broad -nolambda -nomodel. Deeptools (v3.5.4) was used to make a plot of the genome distribution of peaks. The differential analysis of CUT&Tag data utilized the R package DiffBind to find significantly differentially expressed peaks, applying a p-value threshold of 0.05 and a fold change threshold of 2. FastQC was used to evaluate the quality of the ATAC-seq sequencing, and Trim Galore was used to remove low-quality bases and Illumina adaptor sequences from the sequencing runs. Reads with low-quality scores that mapped to the mitochondrial genome and multi-mapped reads were eliminated after the trimmed reads were matched to the bovine reference genome (ARS-UCD1.3) using BWA. MACS2 software was used for peak calling, and the Fraction of Reads in Peak (FRiP) score for each library was determined. For RNA-seq, BGI Tech Solutions Co.‘s HiSeq RNA-Seq was used to examine the total RNA that had been extracted from the designated cells. To minimize sample variance, the RNA from three biological replicas was pooled and blended with an equal amount of RNA in each sample. RNA-Seq transcriptome readings were mapped to the reference genome (hg19) using the Bowtie tool. The RSEM software tool was used to quantify the levels of gene expression, and the possion distribution approach was used to find differentially expressed genes (DEGs).

### ChIP-qPCR assays

Chromatin immunoprecipitations were performed following the manufacturer’s protocol of the Simple ChIP Enzymatic Chromatin IP kit (Cell Signaling Technology, #9003S). In brief, 1% formaldehyde was used to crosslink the cells, then glycine was added to terminate the process. Micrococcal nuclease broke down the chromatin, and the lysate was sonicated with multiple pulses to rupture the nuclear membrane. The processed chromatin was treated with an antibody. Protein G beads were used to separate the complexes. After elution, Proteinase K reversed the cross-links for 2 h at 65 °C. A column was used to purify the released DNA, and qPCR was used for analysis. The primers used are detailed below (5’- > 3’): c-Myc: AAAGAACGGAGGGAGGGATC (Forward), CTATTCGCTCCGGATCTCCC (Reverse); CCND1: CCAAAAGCAAGCAGTGTGGG (Forward), TTAAGCCCTTAAGTCGCCCG (Reverse); CCND2 (5’- > 3’): TCTGTGCAGGATAACACCGAGAC (Forward), CTATCAATGGCAGCGGGAAT (Reverse).

### Tumor sphere formation assays

The sphere formation test media was made by mixing DMEM/F12 with 1 × B27 (Corning), 20 ng/ml EGF (PeproTech), and 10 ng/ml bFGF (PeproTech). 500 μl of the cell suspension was added to each well of ultralow adhesion 24-well plates after the cells had been resuspended in the aforementioned medium at an adjusted density of 1000 cells/ml. Every two days, 200 μl of new media was introduced to each well. For seven days, the spheroids were left to form at 37 °C in a humidified incubator with 5% CO2. Ten days following cell seeding, pictures were taken with a Leica inverted fluorescence microscope.

### Animal experiments

For animal experiments, while no formal statistical methods were applied to calculate sample size, we ensured the inclusion of a sufficient number of animals to observe potential biological effects and account for variability. Simple randomization was used to divide the animals into experimental groups. When conducting animal studies and evaluating the results, the researchers were not blinded to allocation since blinding is not relevant to this study. Pathogen-free male BALB/c nude mice were purchased from the Slaccas in Shanghai. The housing and handling of all mice were conducted in accordance with the authorized procedures of the Ethics Committee of Ruijin Hospital, Shanghai Jiaotong University. For the xenograft lung metastasis tumor model, athemic nude mice were given an injection of FaDU or Cal-27 cells (1×10^6^) into their lateral tail vein. A dissecting microscope was used to estimate the number of metastatic nodules on the lung surface after H&E staining. For the subcutaneous patient-derived xenograft (PDX) models, HNSCC samples were obtained post-surgery from Shanghai Ninth People’s Hospital. All participating patients had been diagnosed with HNSCC and had not undergone any relevant treatments prior to surgery. The acquisition of these samples was made with the informed consent of the patients and received approval from the Ethics Committee. Surgical specimens were promptly preserved in Primary Tissue Storage Solution (Newhunter, HT-PTS) and swiftly transported with ice box to the animal laboratory for nude mice implantation. The tissue was segmented into fragments measuring 1×3×3 mm^3 and surgically engrafted into the subcutaneous tissue of 4-week-old male BALB/c nude mice. These mice were maintained under pathogen-free conditions at Animal laboratory of Shanghai Ruijin Hospital. The weights of the mice and the dimensions of the tumors were documented four times weekly. The tumor volume was calculated using the subsequent formula: Volume (mm^3^) = 0.52 × length (mm) × width (mm) ^2^.

### Statistical analyses

The sample size for this study was determined based on the requirement to ensure adequate statistical power to detect a pre-specified effect size with a significance level (α) of 0.05 and a power (1−β) of 0.8. Power analysis was performed utilizing G*Power software (v3.1.9.7), with effect sizes estimated based on data from previous studies or pilot experiments. Only in cases where sample preparation or data gathering failed were biological samples removed from the study; otherwise, no data were eliminated from the analysis. Samples were allocated to experimental groups by simple randomization for both in vitro and in vivo investigations. The investigators were not blinded to allocation during the experiments and outcome evaluation, as blinding is not pertinent to this investigation. Interpretation of the results was based solely on objective measures and did not rely on subjective evaluation by the authors. As stated in each figure legend, each experiment was conducted two, three, or more times. In addition, statistical analyses in this study were performed using R software (v4.2.2) or GraphPad Prism software (v9.5.1). Estimates of variation within each group are provided as mean ± SD unless otherwise specified and are indicated in figure legends. In general, the two-tailed Student’s t test for normally distributed data or the two-tailed Mann-Whitney test for non-normally distributed data were used to test for differences between the two groups. Wilcoxon rank sum test was used to compare the difference of ranked data in two groups. Tukey’s multiple comparisons test and two-way analysis of variance (ANOVA) were used to assess the numerous comparisons. Additionally, Sidak’s post hoc test was used in conjunction with a two-way repeated-measures ANOVA to evaluate differences in time-course experiments. The Pearson correlation coefficient was used for correlation analysis, while the Log-rank test was used for survival analysis. A *P*-value less than 0.05 was deemed significant.

## Supplementary information

Supplementary Figure Legends

Figure S1

Figure S2

Figure S3

Figure S4

Figure S5

Figure S6

Uncropped images of blots

## Data Availability

The authors confirm that the data supporting the findings of this study are available within the article and its supplementary materials, or available from the corresponding author on reasonable request. The sequencing data generated in this study have been deposited in the NCBI’s Sequence Read Archive (SRA) under the accession number of PRJNA1205878. All other raw data are available upon request from the corresponding author.
