# Supplementary material for: USP10 stabilizes BAZ1A to drive tumor stemness via an epigenetic mechanism in head and neck squamous cell carcinoma
Source: Cell Death Dis. 2025 Apr 10;16(1):270. doi: 10.1038/s41419-025-07462-x (PMC11982335; doi:10.1038/s41419-025-07462-x)
Supplement: Supplementary file 1 — Supplementary Figure Legends [file 41419_2025_7462_MOESM1_ESM.docx]

**Supplementary figure legends**

**Figure S1 USP10 enhances HNSCC proliferation and growth in virto and in vivo. (A)** Western blotting and RT-qPCR assays showing the USP10-high and -low HNSCC cell lines. **(B)** Western blotting assays showing the USP10 levels in control and USP10-OE Cal-33, or FaDU cells. **(C)** Western blotting assays showing the USP10 expressions in parental and USP10-KO Cal-27, or SCC-15 cells. **(D)** CCK-8 analysis showing the proliferation rates of indicated cells. **(E)** Colony formation assays showing the growth of Cal-33, or FaDU cells transfected with or without USP10-CA plasmids. **(F-G)** EdU staining assays were conducted to assess the proliferation ability of HNSCC cells. **(H)** Comparison of tumor weights in mice from indicated groups. **(I)** Kaplan-Meir survival curves of mice from Figure 2(G). Data represent the Mean±SD of at least three independent experiments. *P < 0.05, **P < 0.01, and ***P < 0.001. Differences were tested using an un-paired Student’s t-test (A, E-H), the 2-way ANOVA followed by Tukey’s multiple comparisons test (D), and the log-rank test (I).

**Figure S2 USP10-BAZ1A axis regulates HNSCC tumor stemness. (A-B)** Transwell assay (A) of Cal-27 and SCC-15 cells with or without USP10 depletion, respectively. Right panel (B) shows the quantification of transwell assay results. **(C)** Wound-healing assays were shown in control and USP10-KO Cal-27 cells. **(D)** Wound-healing assays were shown in FaDU cells with or without USP10 overexpression. **(E)** Western blotting assays showing the expressions of EMT-related markers in Cal-27 and SCC-15 cells with or without USP10 knockdown. (F) Quantification data of lung metastasis rates in mice derived from Figure 3(D, G). *P < 0.05, **P < 0.01, and ***P < 0.001. Differences were tested using an un-paired Student’s t-test (B-D).

**Figure S3 USP10 depends on BAZ1A to enhance HNSCC stemness and progression. (A)** Tandem affinity purification of USP10-related protein complex was conducted using Cal-27 cells stably expressing Flag-HA-USP10. Associated proteins were separated by SDS-PAGE and visualized by CB staining (A). The number of total peptides identified by mass spectrometry analysis is shown in right. **(B)** Boxplot showing the expressions of BAZ1A in tumor and normal samples from TCGA-HNSCC. **(C)** RT-qPCR analysis showing the BAZ1A expressions in cells transfected with shRNAs. **(D)** Colony formation and transwell assays were conducted in indicated cells. **(E)** Volcano plot showing the distributions of DEGs, where red represents the up-regulated genes, and blue represents the down-regulated genes. **(F)** RT-qPCR analysis showing the mRNA levels of CSC-related genes in cells transfected with EV or BAZ1A-δBrd mutants. **(G)** Heatmap showing BAZ1A-signature genes that are differentially expressed between low- and high-stemness HNSCC (source: TCGA-HNSCC). **(H)** Correlation between BAZ1A-signature t score and mRNAsi value. **(I)** BAZ1A-signature t scores stratify patients with HNSCC (by K-mean clustering) into high-risk (t score > 0) and low-risk groups (t score % 0) for survival rate. **(J)** Tumor sphere formation assays were conducted in the indicated cells. Quantification data was shown on the right. **(K)** Western blot showing the levels of stemness markers (CD44, CD133) in control and USP10-overexpressing HNSCC cells. **(L)** Western blot showing the levels of CD44, and CD133 in control and USP10-KD Cal-27 cells. Data represent the Mean±SD of at least three independent experiments. *P < 0.05, **P < 0.01, and ***P < 0.001. Differences were tested using an un-paired Student’s t-test (B-C, F, J), and the log-rank test (I).

**Figure S4 BAZ1A regulates CSC-related genes via epigenetic remodeling. (A)** A summary of BAZ1A fragments interacting with SOX2 is shown. **(B)** CCND1-, c-Myc and Survivin-promoter-driven luciferase activities were assessed in indicated cells. **(C)** Gene tracks of CUT&Tag-seq signal for BAZ1A, SOX2, and H3K27ac at the c-Myc gene. The x-axis: Genomic position of promoter regions (P), or enhancer (E). The y-axis: CUT&Tag-seq signal (reads per million per base pair). **(D)** dCas9-KRAB based CRISPR interference (CRISPRi) is utilized to specifically silence enhancer by enhancer specific sgRNA. ChIP-qPCR of BAZ1A binding in promoter or enhancer of CCND1 and c-Myc genes (upper). RT-qPCR analysis of mRNA levels for CCND1, c-Myc was conducted (lower). Data represent the Mean±SD of at least three independent experiments. *P < 0.05, **P < 0.01, and ***P < 0.001. Differences were tested using an unpaired Student’s t-test (B, D).

**Figure S5 BAZ1A inhibitor is effective to enhance cisplatin efficacy in HNSCC. (A)** Western blot analysis of USP10-BAZ1A-CCND1 protein levels in Cal-27 and SCC-15 cells with or without cisplatin resistance. **(B-C)** Mouse weight change measurements (B) (n=5) and complete blood counts (C) were performed on vehicle control and BAZ1A-IN-1 treated mice. **(D)** The qRT-PCR analysis of CSC-characteristic genes in two paired HNSCC-PDX models. **(E)** The IHC images showing the representative markers (Ki-67, CCND1, c-Myc, EpCAM) in PDX tumors from indicated groups in Figure 7(H). Data represent the Mean±SD of at least three independent experiments. *P < 0.05, **P < 0.01, and ***P < 0.001. Differences were tested using an unpaired Student’s t-test (C-D).
