# Supplementary figures and images for: USP10 stabilizes BAZ1A to drive tumor stemness via an epigenetic mechanism in head and neck squamous cell carcinoma
Source: Cell Death Dis. 2025 Apr 10;16(1):270. doi: 10.1038/s41419-025-07462-x (PMC11982335; doi:10.1038/s41419-025-07462-x)

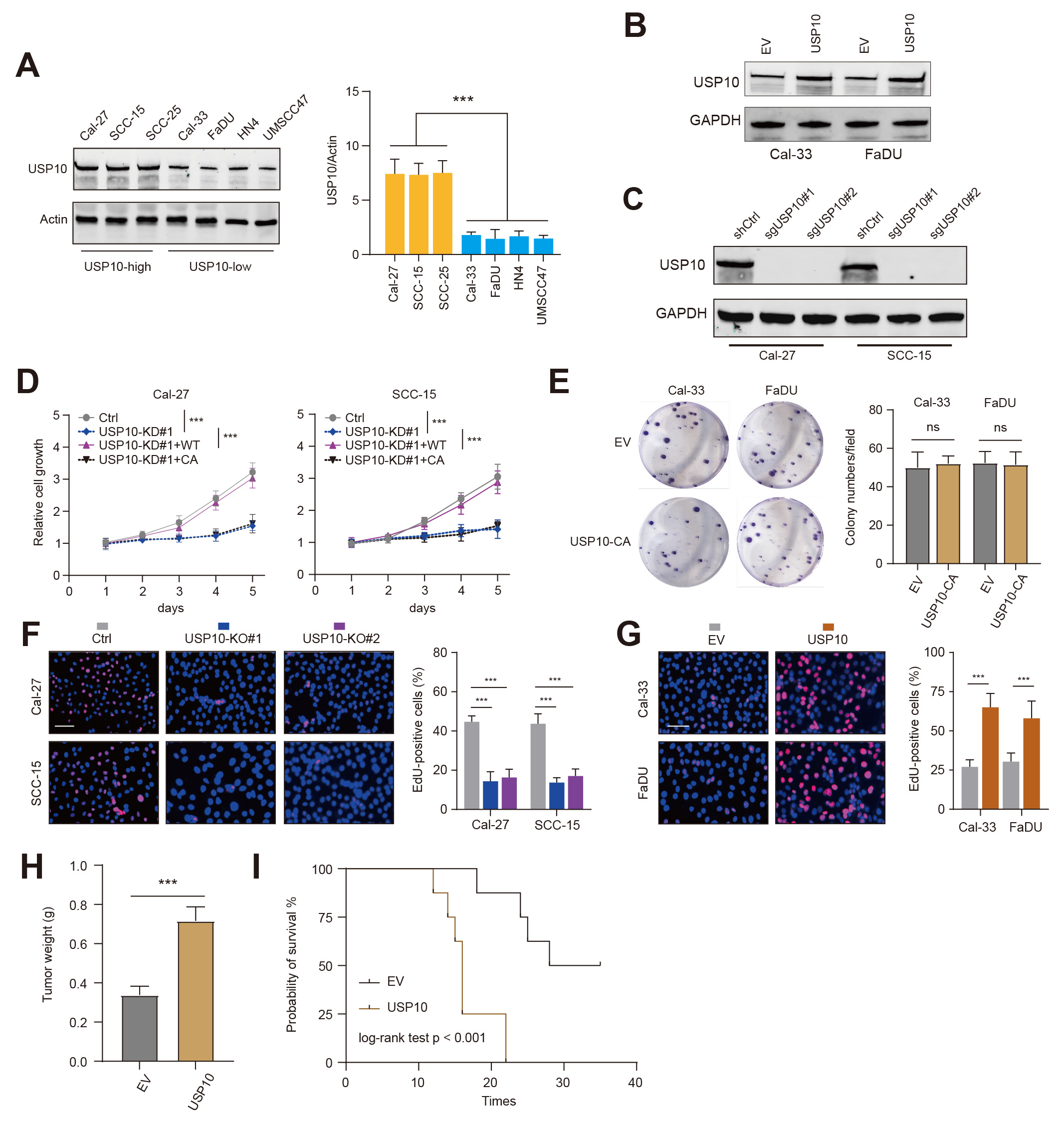

Supplement: Supplementary file 2 — Figure S1 [file 41419_2025_7462_MOESM2_ESM.jpg]

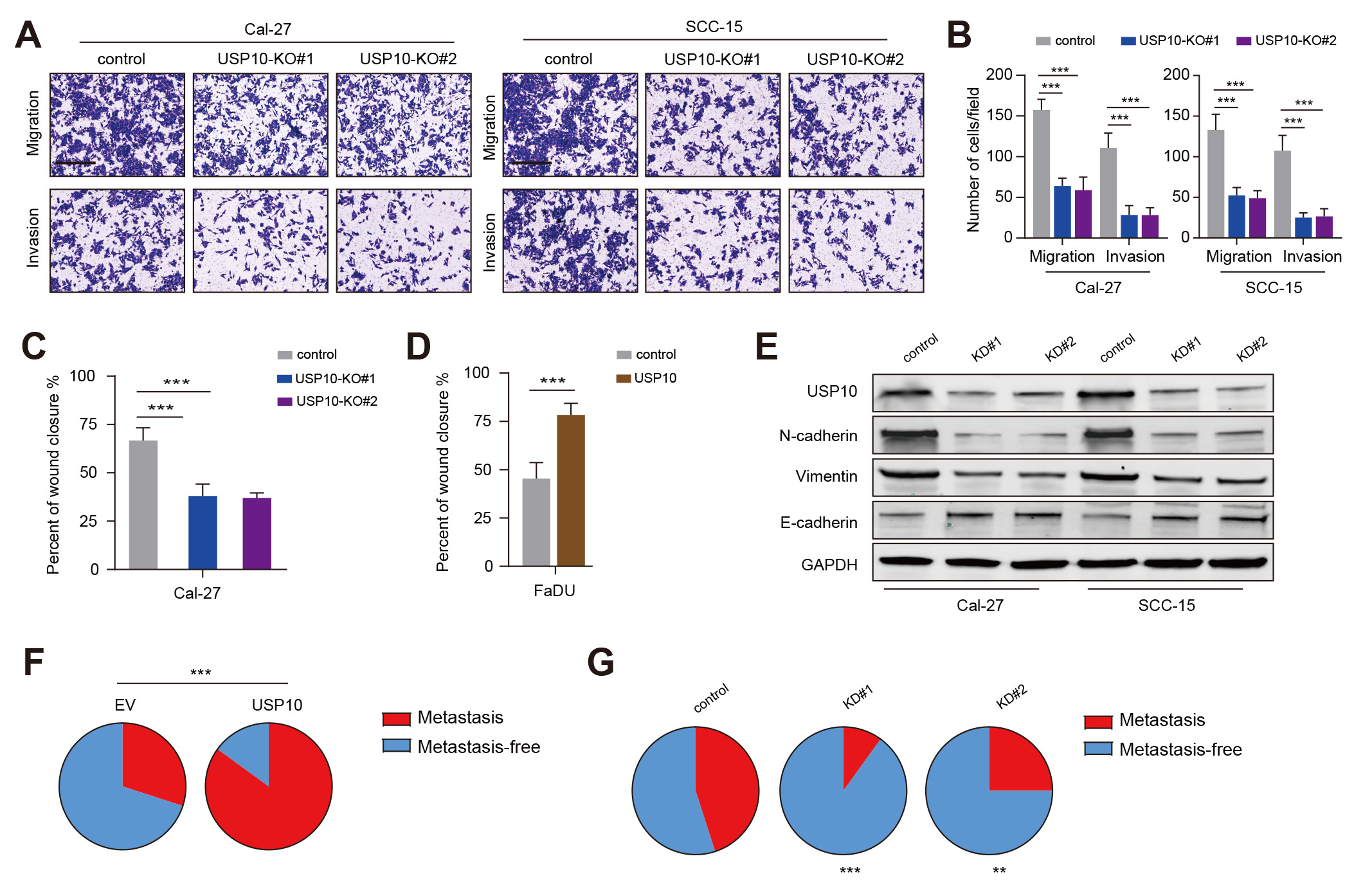

Supplement: Supplementary file 3 — Figure S2 [file 41419_2025_7462_MOESM3_ESM.jpg]

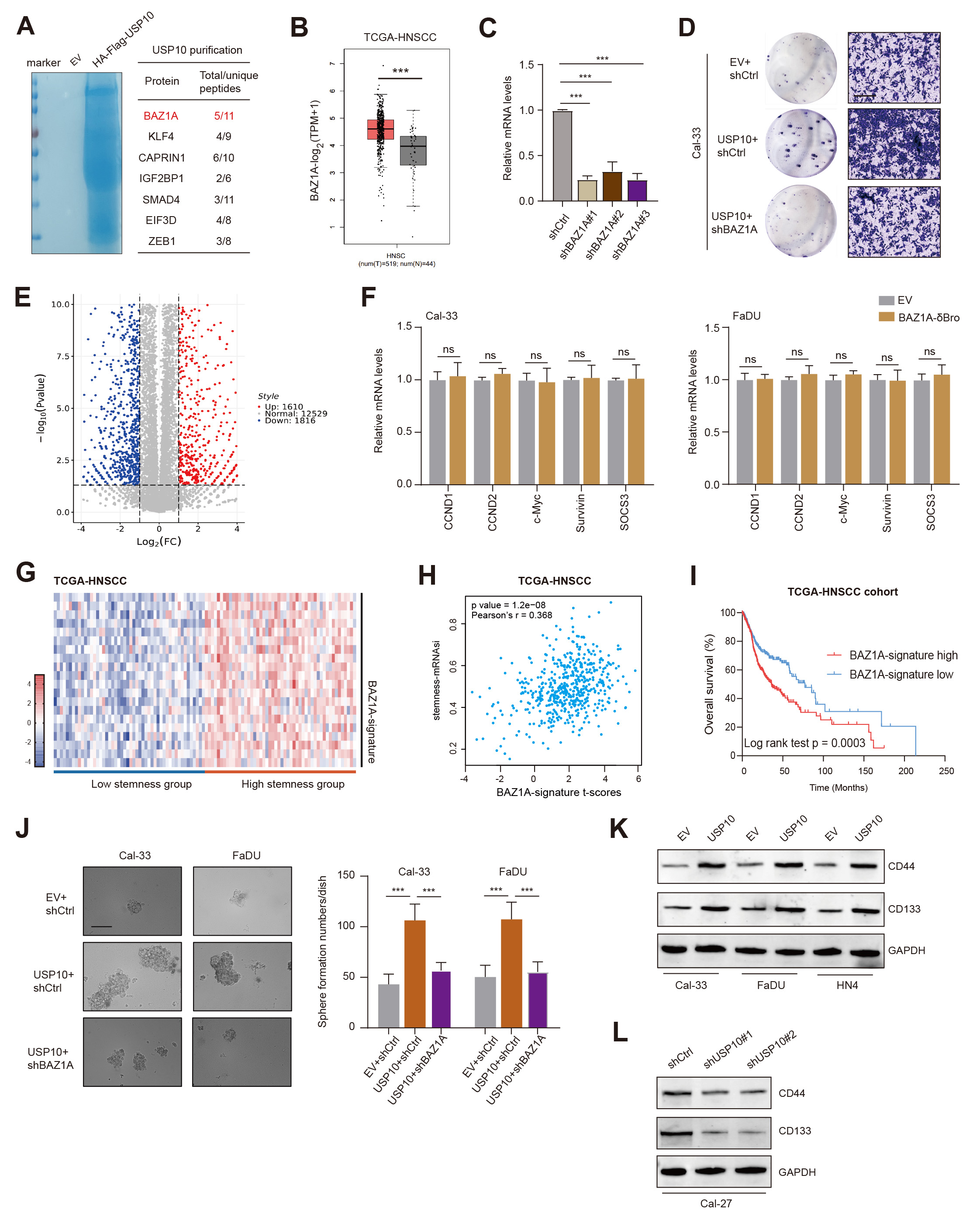

Supplement: Supplementary file 4 — Figure S3 [file 41419_2025_7462_MOESM4_ESM.jpg]

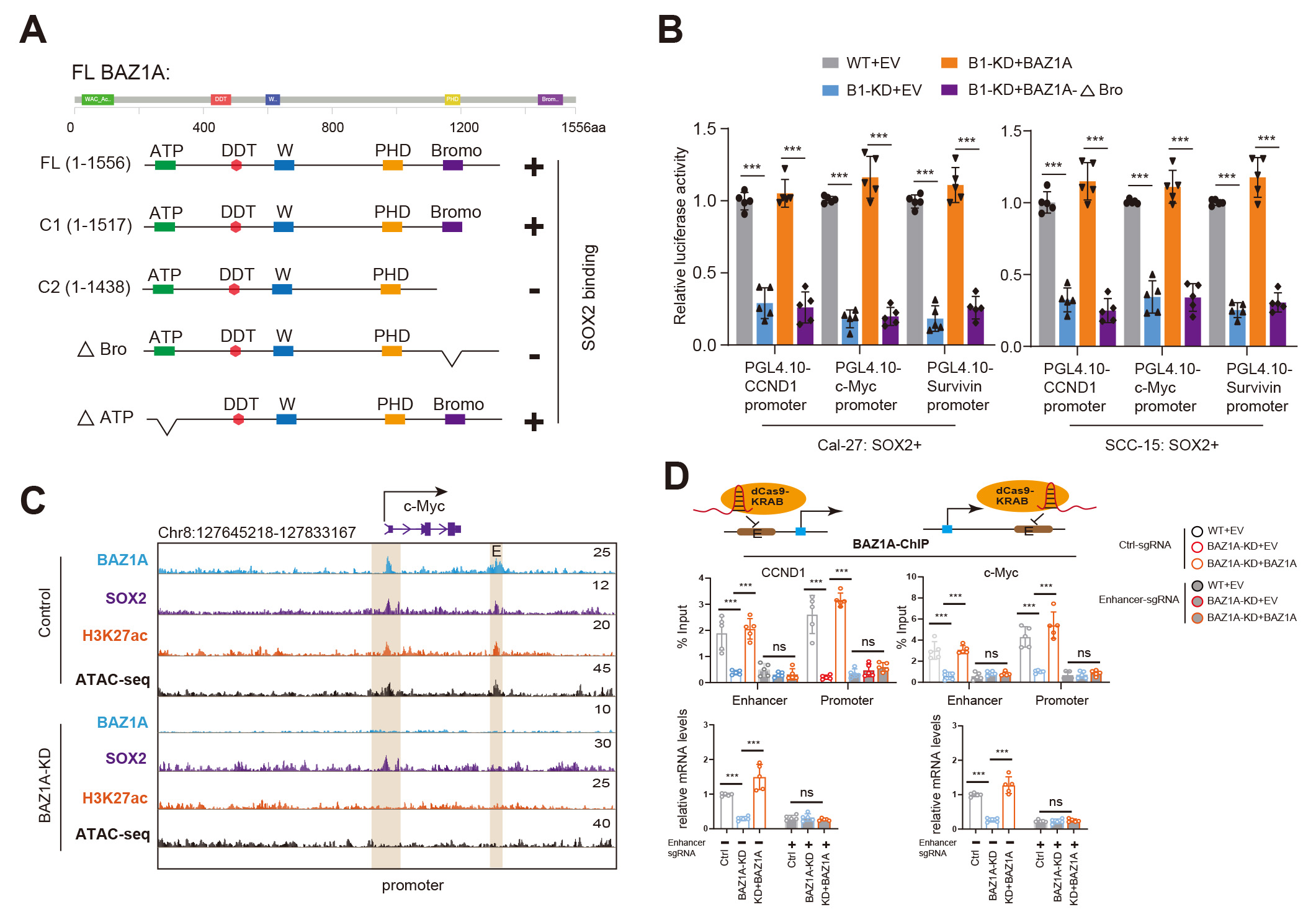

Supplement: Supplementary file 5 — Figure S4 [file 41419_2025_7462_MOESM5_ESM.jpg]

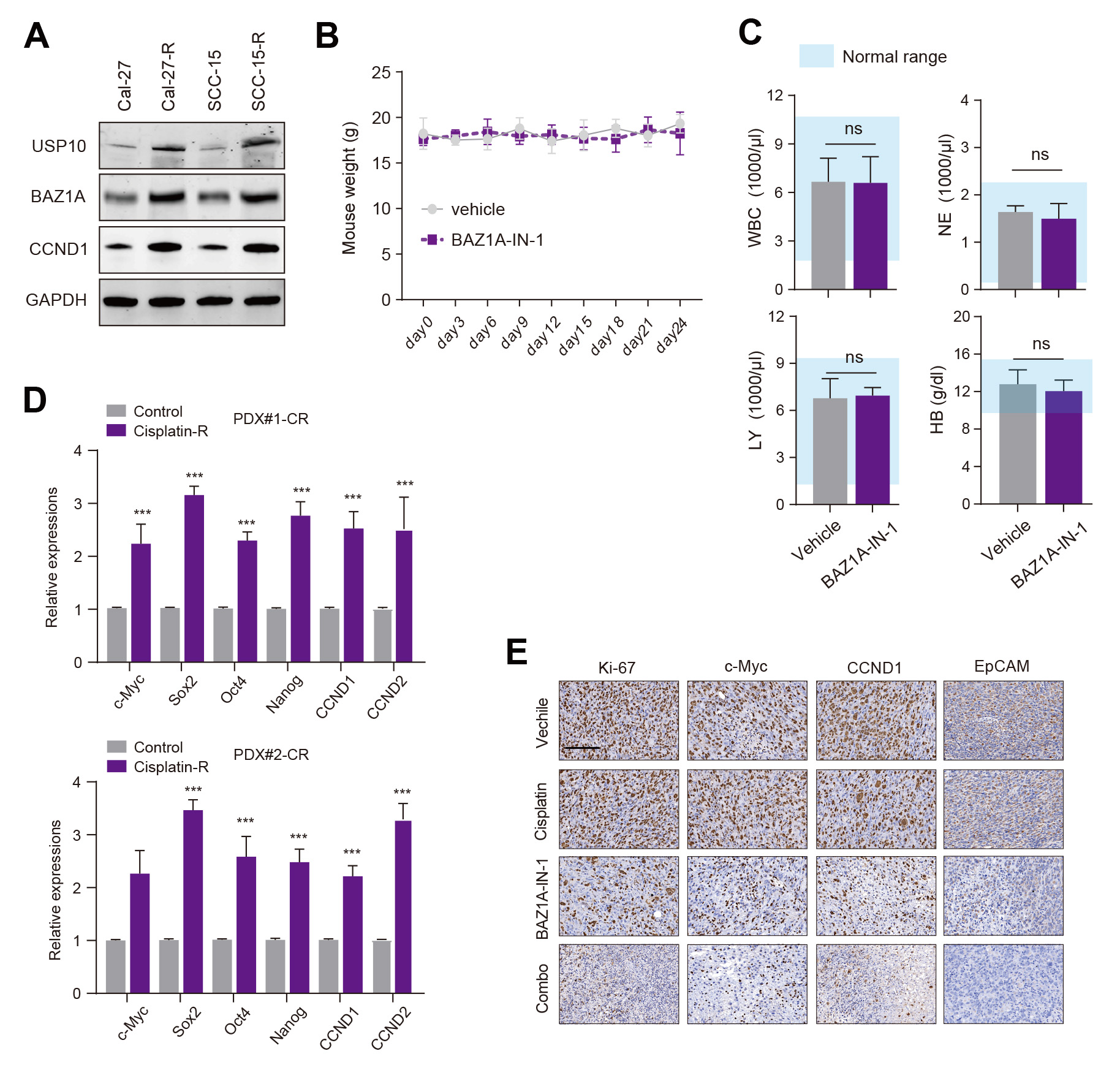

Supplement: Supplementary file 6 — Figure S5 [file 41419_2025_7462_MOESM6_ESM.jpg]

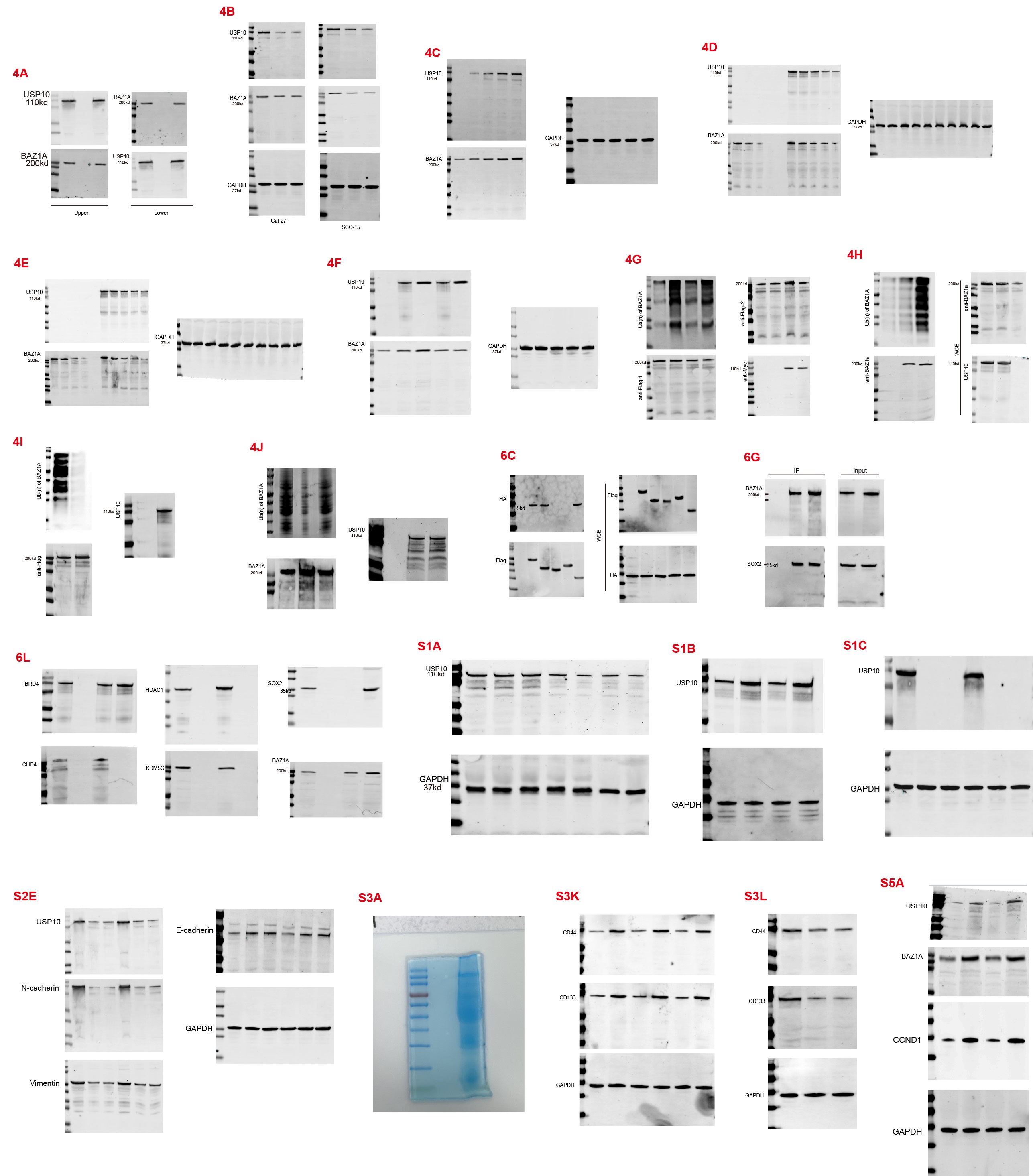

Supplement: Supplementary file 7 — Figure S6 [file 41419_2025_7462_MOESM7_ESM.jpg]

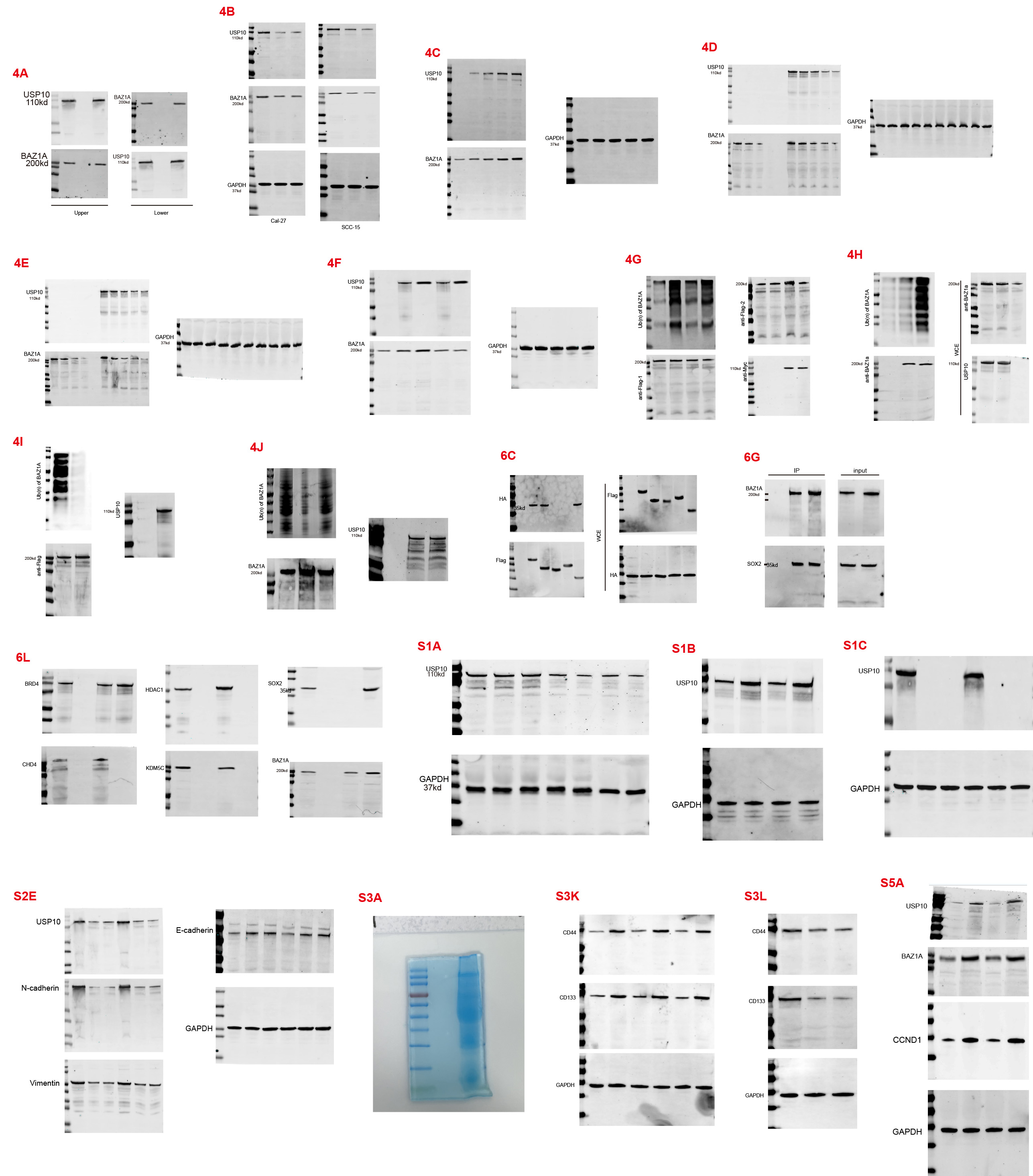

Supplement: Supplementary file 8 — Uncropped images of blots [file 41419_2025_7462_MOESM8_ESM.jpg]
